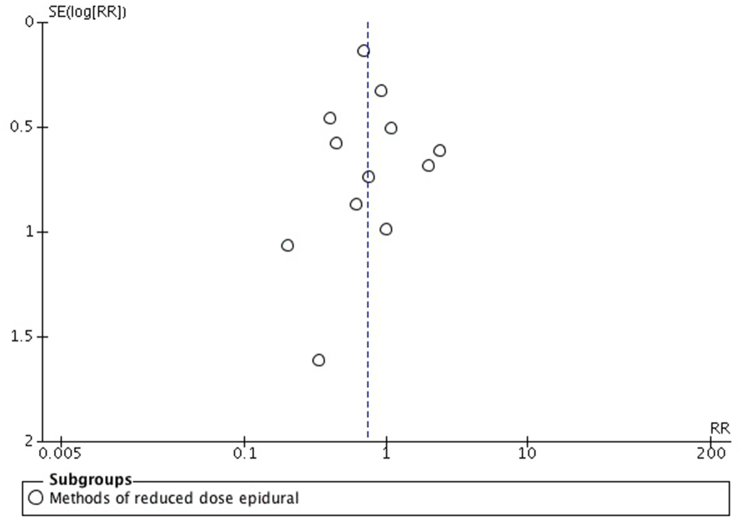# Interventions for the prevention or treatment of epidural-related maternal fever: a systematic review and meta-analysis

**DOI:** 10.1016/j.bja.2022.06.022

**Published:** 2022-08-05

**Authors:** Anna Cartledge, Daniel Hind, Mike Bradburn, Marrissa Martyn-St James, Sophie Davenport, Wei Shao Tung, Hwu Yung, Jeyinn Wong, Matthew Wilson

**Affiliations:** 1 School of Health and Related Research, University of Sheffield, Sheffield, UK; 2 The Medical School, University of Sheffield, Sheffield, UK; 3 General Surgery, Nottingham University Hospitals NHS Trust, Trust Headquarters, Nottingham, UK

**Keywords:** epidural fever, epidural-related maternal fever, intrapartum fever, labour analgesia, labour epidural, perinatal analgesia

## Abstract

**Background:**

Epidural-related maternal fever in women in labour has consequences for the mother and neonate. There has been no systematic review of preventive strategies.

**Methods:**

RCTs evaluating methods of preventing or treating epidural-related maternal fever in women in active labour were eligible. We searched MEDLINE, EMBASE, CINAHL, Web of Science, CENTRAL, and grey literature sources were searched from inception to April 2021. Two review authors independently undertook study selection. Data extraction and quality assessment was performed by a single author and checked by a second. The Cochrane Risk of Bias 2 tool was used. Meta-analyses for the primary outcome, incidence of intrapartum fever, were performed using the DerSimonian and Laird random effects model to produce summary risk ratios (RRs) with 95% confidence intervals (95% CIs).

**Results:**

Forty-two records, representing 34 studies, were included. Methods of reduced dose epidural reduced the incidence of intrapartum fever, but this was not statistically significant when six trials at high risk of bias were removed (seven trials; 857 participants; RR=0.83; 95% CI, 0.41–1.67). Alternative methods of analgesia and high-dose prophylactic systemic steroids reduced the risk of intrapartum fever compared with epidural analgesia. Prophylactic paracetamol was not effective.

**Conclusions:**

There is no clear evidence to support the use of any individual preventative or therapeutic intervention for epidural-related maternal fever. Further research should focus on understanding the mechanism of fever development to enable RCTs of potential interventions to reduce the incidence of intrapartum fever development and the subsequent disease burden felt by the neonate.

**Clinical trial registration:**

CRD42021246929.

**Editor's key points**

• Epidural analgesia is a cause of fever. A previous systematic review showed that intrapartum fever of any cause is associated with neonatal brain injury.
• This systematic review of 34 studies indicates that there is no clear evidence to support the use of any individual preventative or therapeutic intervention for epidural-related maternal fever.
• Mechanistic research is required to understand how fever develops after epidural analgesia. This will support the development and evaluation of targeted interventions.

Fever in labour is common and may be attributable to infective causes, the use of epidural analgesia, or environmental factors such as heated delivery rooms.^1^ Over the past two decades epidural analgesia has become a more popular choice; rates range from 10% to 83%, consistently higher in nulliparous women.^2^ In labouring women who receive epidural analgesia 15–25% will develop a sterile clinical fever.^3^^,^^4^ Epidural-related maternal fever (ERMF) is specifically observed in labouring women, and not seen in non-pregnant women or even pregnant women who receive an epidural for an elective Caesarean delivery.^3^^,^^5^

**Table 1:** Summary of findings for the primary outcome, incidence of intrapartum fever. The risk in the intervention group (and its 95% confidence interval) is based on the assumed risk in the comparison group and the relative effect of the intervention (and its 95% CI). ∗Downgraded one level owing to study limitations: high risk of bias. ^†^Downgraded one level because of study limitations: no study assessed as being low risk of bias. Issues with lack of blinding and crossover in three trials. ^‡^Downgraded one level owing to imprecision: 95% CI includes null effect. CI, confidence interval; GRADE, Grading of Recommendations Assessment, Development, and Evaluation; RR, risk ratio.

Intervention type	No of participants (studies)	Certainty of the evidence (GRADE)	Relative effect (95% CI)	Anticipated absolute effects	
				Risk with trial intervention	Risk difference trial intervention
Reduced dose epidural	4164 (12 RCTs)	⨁⨁⨁◯ MODERATE∗	RR=0.74 (0.610–0.92)	91 per 1000	24 fewer per 1000 (36 fewer to 7 fewer)
Alternative methods of analgesia	2163 (8 RCTs)	⨁⨁⨁◯ MODERATE^†^	RR=0.46 (0.32–0.66)	141 per 1000	76 fewer per 1000 (96 fewer to 48 fewer)
Prophylactic steroids	270 (3 RCTs)	⨁⨁⨁⨁ HIGH	RR=0.19 (0.05–0.71)	161 per 1000	131 fewer per 1000 (153 fewer to 47 more)
Prophylactic paracetamol	221 (3 RCTs)	⨁⨁⨁◯ MODERATE^‡^	RR=0.71 (0.33–1.53)	126 per 1000	37 fewer per 1000 (85 fewer to 67 more)

Despite ERMF first being identified in 1989,^6^ the underlying causative pathway remains largely unclear. Various mechanisms have been proposed including thermoregulatory disruption by sympathetic blockade, systemic opioid administration dampening fever development in those who elect not to receive epidural, and more recently inflammatory processes.^6^

Currently, the two main candidate mechanisms supported by research evidence are inhibition of cutaneous heat loss^7^ and ‘sterile’ inflammation from exposure to continuous infusion of local anaesthetic mediated by elevation in pro-inflammatory cytokines, for example interleukin-6 (IL-6) at a cellular level.^4^^,^^8^, ^9^, ^10^

A range of preventative interventions have been evaluated in the management of ERMF, based on different assumptions of the underlying mechanism, including intermittent administration of epidural analgesia, compared with continuous infusion,^11^^,^^12^ to reduce local anaesthetic dose, prophylactic steroids,^13^ and prophylactic paracetamol.^14^ The use of antibiotic prophylaxis has also been investigated to explore whether infection is a cause of ERMF, and whether antibiotics are effective in reducing its incidence.^15^

ERMF is an under-researched problem; previous systematic reviews focused on confirming the association between epidural analgesia and the development of maternal fever,^16^^,^^17^ or evaluating the consequences of fever on the mother and neonate.^17^^,^^18^ Another systematic review compared the use of intravenous remifentanil as an alternative to epidural analgesia,^19^ but methods of preventing or treating fever when an epidural is already placed, or is inevitable because of analgesia requirements, have not been systematically reviewed. The aim of this systematic review is to evaluate the RCT evidence of the comparative effectiveness of interventions to prevent or treat the development of ERMF.

Our primary outcome was the overall incidence of ERMF, however defined by the individual studies. Secondary outcomes were the incidence of neonatal sepsis evaluation and incidence of neonatal admission to Level 2 care. Inflammatory markers, when reported, were also evaluated.

## Methods

This systematic review was reported in accordance with the Preferred Reporting Items for Systematic Reviews and Meta-Analyses (PRISMA) 2020 statement.^20^ A protocol was registered on PROSPERO before study selection was undertaken (CRD42021246929).^21^

### Eligibility criteria

Randomised controlled trials that evaluated methods of preventing or treating ERMF were eligible for inclusion. For preventative strategies, inclusion criteria included studies that examined women in spontaneous or induced active labour, that evaluated methods including, but not limited to, alternative methods of analgesia, methods of reduced epidural dosage, and prophylactic paracetamol or steroids. For therapeutic strategies, included studies evaluated methods including, but not limited to, steroid, paracetamol, or antibiotics, if administered on identification or fever. Epidural analgesia had to include a local anaesthetic component administered into the epidural space. Trials that evaluated spinal anaesthesia only or did not include any local anaesthetic in the epidural solution were excluded. Eligibility was not restricted based on comparator intervention.

### Search strategy

MEDLINE, EMBASE, CINAHL, Web of Science, and CENTRAL were searched from inception to search date (April 8, 2021) using a combination of MeSH headings and free text terms. No language, date, or publication limits were applied to the search. The Society of Obstetric Anaesthesia and Perinatology, the Society for Maternal Foetal Medicine, the Royal College of Obstetrics and Gynaecology, and the Obstetric Anaesthetists Association were hand searched using the key words ‘epidural related fever’, ‘intrapartum fever’, and ‘maternal fever’. One reviewer hand-searched the reference lists and performed citation tracking in Google Scholar of all included studies to ensure literature saturation. The final search strategy is available in Supplementary file 1.

### Study selection

Two review authors (AC and SD) independently performed study selection in two stages. Firstly, the titles and abstracts of all publications were screened, and then full texts of records that appeared to meet the eligibility criteria were retrieved and screened for inclusion. Reasons for excluding articles based on review of the full text was recorded. Disagreements were resolved through discussion with a third reviewer (DH).

### Data extraction

AC independently extracted data into a predetermined data extraction sheet. Data on study characteristics (author, year, country, study design, funding source, eligibility and exclusion criteria, intervention type, sample size, and whether intention-to-treat analysis was performed), population characteristics (age, parity, gestation, whether labour was spontaneous or induced, baseline temperature, and baseline cervical dilation), intervention and comparator characteristics (method of epidural initiation if applicable, dose and frequency of primary intervention, patient-controlled analgesia, description of any additional intervention, any modifications), description of outcome definitions (definition of fever, guidelines for neonatal sepsis evaluation), and study results for the review outcomes were extracted. A second reviewer checked the accuracy of data extraction of the primary outcome on all included studies (DH), and for all other data items (SD) on a 20% sample of included studies. Three Chinese-speaking review authors (WT, HY, JW) independently extracted the data from studies that did not have an English full text available, and any discrepancies were discussed with a fourth author (AC).

### Risk of bias

A single review author (AC) assessed the risk of bias in included studies using the Cochrane Risk of Bias 2 (RoB 2) tool,^22^ with all attributions checked by a second (DH) and, in some cases a third author (MW). Following the comprehensive guidance and signalling questions,^23^ each domain was graded as ‘low’, ‘some concerns’, or ‘high’ risk. The grading in each domain was used to inform the overall judgment of the risk of bias in each study.

### Data synthesis and analysis

When two of three arms were for different doses of a therapeutic intervention, the highest dose was included in the meta-analysis. In the case of trials with multiple arms evaluating dose reduction strategies for the local anaesthetic component of epidural analgesia, the lowest dose arm was included in the meta-analysis.

Meta-analyses were carried out in RevMan 5,^24^ using the DerSimonian and Laird inverse variance random effects model. Heterogeneity was assessed using the *I*^2^ statistic, but this did not influence decisions to perform subgroup or sensitivity analyses. Publication and small study biases were examined using both funnel and Egger plots^25^ generated by the Stata statistical software.^26^ Risk ratios (RRs) and 95% confidence intervals (95% CIs) were used for binary outcomes.

Because of intervention heterogeneity, no overall analysis was undertaken. Instead, meta-analyses grouped studies by the type of intervention, with subgroup analysis performed where there was variation within intervention categories and ≥3 studies evaluated comparable interventions with similar comparators.

The quality of evidence for each prespecified outcome reported by at least one study in each comparison was assessed using the GRADEpro approach.^27^^,^^28^ AC performed the initial GRADE (Grading of Recommendations Assessment, Development, and Evaluation) analysis, and this was verified by MW.

## Results

### Study selection

**Fig 1:**
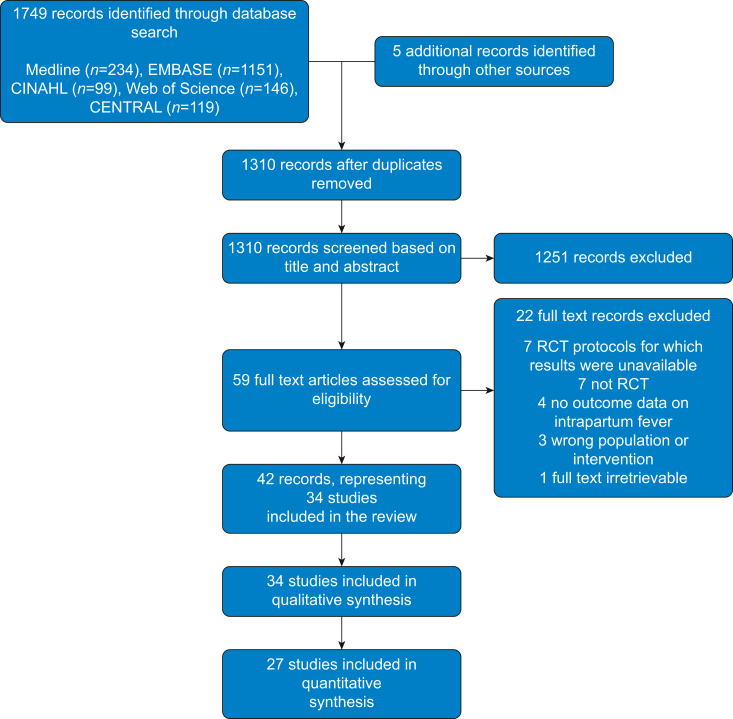
Preferred Reporting Items for Systematic Reviews and Meta-Analyses (PRISMA) flow diagram.

A total of 1749 records were identified by the electronic database search, and 1310 titles and abstracts were screened after de-duplication (Fig 1). Fifty-nine records were screened at full text, of which 37 were eligible for inclusion. Reasons for exclusion at full text included non-RCTs,^29^, ^30^, ^31^, ^32^, ^33^, ^34^, ^35^ results not being available,^36^, ^37^, ^38^, ^39^ incorrect population or intervention,^40^, ^41^, ^42^ or no outcome data on the incidence of intrapartum fever.^43^, ^44^, ^45^, ^46^ A further four excluded records were trial protocols for eligible RCTs but had no published results.^47^, ^48^, ^49^, ^50^ The authors of these trials were contacted and two responded confirming the trials were still ongoing in June 2021 and so were unable to provide any results^48^^,^^50^ and two did not respond.^47^^,^^49^

An additional four records were identified by searching the grey literature, and one final record was obtained by correspondence with a trial author (P. Steer, personal communication, 2021). Thus, a total of 42 records^11^, ^12^, ^13^, ^14^, ^15^^,^^51^, ^52^, ^53^, ^54^, ^55^, ^56^, ^57^, ^58^, ^59^, ^60^, ^61^, ^62^, ^63^, ^64^, ^65^, ^66^, ^67^, ^68^, ^69^, ^70^, ^71^, ^72^, ^73^, ^74^, ^75^, ^76^, ^77^, ^78^, ^79^, ^80^, ^81^, ^82^, ^83^, ^84^, ^85^, ^86^, ^87^ – representing 34 studies and including a total 10 221 participants – are included in the review.

### Characteristics of included studies

All included trials were parallel-group, participant-RCTs. Thirty were single centre; the remaining four multicentre.^57^^,^^60^^,^^61^^,^^77^ The sample size ranged from 42^14^ to 3000,^12^ but in general studies were small with 26 of the 34 studies having 200 participants or fewer. A table of the characteristics on individual studies is included in Supplementary file 2.

### Population characteristics

A table of baseline population characteristics is included in Supplementary file 3. The average age of participants ranged from 21^13^^,^^14^^,^^69^ to 32^64^ yr, with no studies including women aged less than 18 yr. Only 15 studies reported the average baseline temperature of participants, all of which were lower than 37.5°C. A potential important source of heterogeneity between study populations is the mean cervical dilation of participants at baseline as this ranged considerably from 1.8^65,67^ to 5.0 cm.^13^^,^^15^^,^^69^ Ten studies involved participants with a mean or median, however reported, cervical dilation ≥4 cm at baseline^13^^,^^14^^,^^55^^,^^57^^,^^60^^,^^64^^,^^69^^,^^77^^,^^78^ and three reported participants had a baseline dilation of <2 cm.^12^^,^^51^^,^^65^^,^^67^ No study raised concerns regarding imbalances in participant characteristics at baseline.

### Intervention characteristics

Only studies evaluating methods of preventing the development of ERMF were identified. Thirteen studies evaluated various methods of reducing epidural dose: intermittent administration,^11^^,^^12^^,^^80^ lower dose formulations,^56^^,^^67^^,^^83^ automated bolus regimens,^66^^,^^68^^,^^73^^,^^74^ delayed administration,^71^ reduced rate administration,^52^ or local anaesthetic only with no spinal analgesia component.^72^ Eight compared alternative methods of analgesia,^54^^,^^57^^,^^60^^,^^64^^,^^69^^,^^77^^,^^78^^,^^82^ one evaluated additional epidural opioid,^65^ two trials evaluated either warming the epidural solution^59^ or using a heated neck warmer,^74^ and two trials evaluated methods of acupuncture.^51^^,^^55^ Three trials evaluated steroid^13^^,^^85^^,^^87^ prophylaxis, two trials evaluated paracetamol^14^^,^^58^ prophylaxis, and one trial evaluated antibiotic^15^ prophylaxis. One trial evaluated both intravenous patient-controlled analgesia (PCA) remifentanil and prophylactic paracetamol.^76^ Finally, one trial evaluated delayed initiation of analgesia compared with routine epidural analgesia at the start of labour.^61^

### Comparator characteristics

Because of the variability of intervention, the comparator intervention depended on the intervention under study. The comparator in 24 of the 34 studies was an active control arm. This was either a different dose,^56^^,^^67^^,^^84^ drug,^54^^,^^57^^,^^60^^,^^64^^,^^65^^,^^69^^,^^78^^,^^79^ or administration process^11^^,^^12^^,^^52^^,^^59^^,^^61^^,^^66^^,^^68^^,^^71^^,^^75^^,^^82^ to the trial intervention. In four trials all participants received the same epidural analgesia and an additional trial intervention that had no active comparator.^51^^,^^55^^,^^76^^,^^83^^,^^87^ Placebos were used by the trial evaluating prophylactic antibiotics,^15^ two trials evaluating prophylactic steroids^13^^,^^85^ and two trials evaluating prophylactic paracetamol.^14^^,^^58^

### Risk of bias

**Fig 2:**
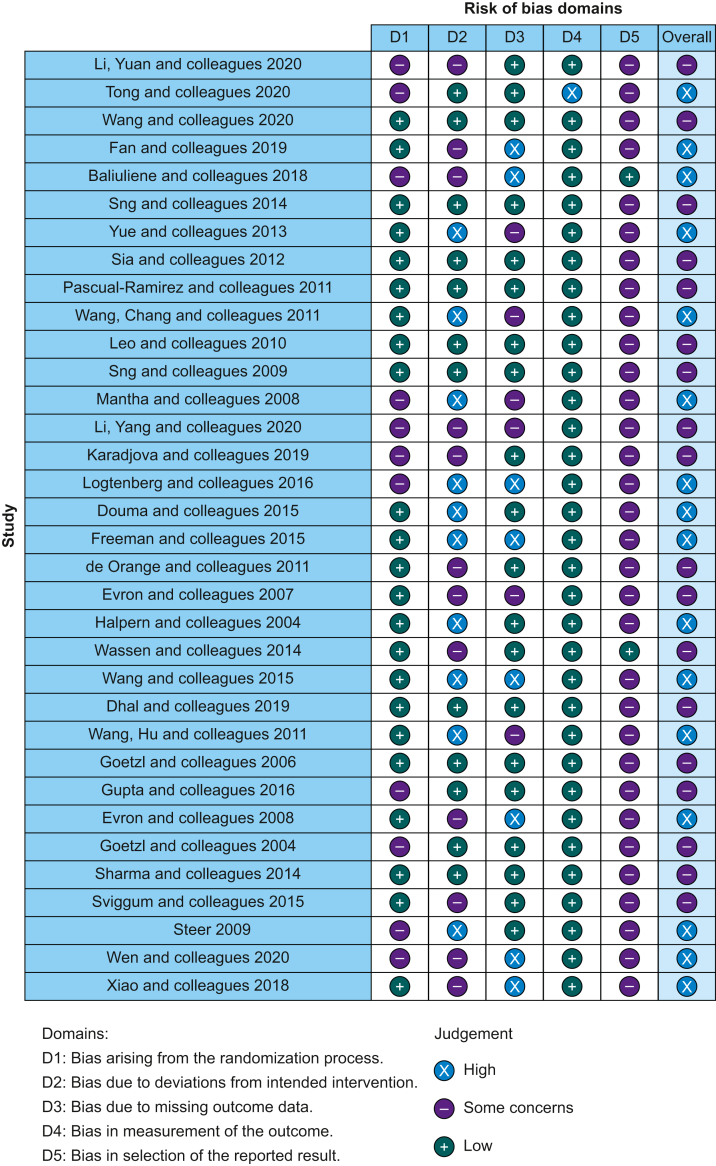
Risk of bias of individual studies.

The risk of bias across studies was variable (Fig 2). Full explanations for the risk of bias judgements are included in Supplementary file 4. No studies were assessed as having a low risk of bias.

Thirty trials reported appropriate allocation sequence generation, using either a computer-based random number generator or random number tables, or a web-based randomisation programme, and the remaining four trials did not provide information on the randomisation.^54^^,^^60^^,^^61^^,^^74^ Twenty-one trials concealed the allocation sequence until participants were assigned to intervention by using sequentially numbered, opaque, sealed envelopes, and two trials^13^^,^^15^ reported using identical preparations of the study drugs. Ten trials did not provide any information on allocation concealment^11^^,^^14^^,^^51^^,^^52^^,^^54^^,^^56^^,^^60^^,^^74^^,^^82^^,^^83^ and one trial stated that allocation was not concealed at all.^57^ Twenty trials stated that participants were blinded.^12^, ^13^, ^14^, ^15^^,^^52^^,^^56^^,^^59^^,^^66^, ^67^, ^68^^,^^72^^,^^73^^,^^75^^,^^76^^,^^78^^,^^83^, ^84^, ^85^, ^86^, ^87^ The clinical teams and clinicians delivering the intervention were blinded in 15.^12^^,^^13^^,^^52^^,^^56^^,^^58^^,^^59^^,^^67^^,^^68^^,^^72^^,^^76^^,^^83^, ^84^, ^85^^,^^87^ It was unclear whether participants and clinical teams were blinded in one^82^ and six^14^^,^^15^^,^^66^^,^^75^^,^^78^^,^^82^ trials, respectively. In the remaining 13 trials, all participants and clinical teams were aware of the assigned intervention groups.

Trials did not perform intention-to-treat analysis, and therefore suffered attrition bias, if participants were excluded after randomisation; usually if they delivered via Caesarean section or within 2 h of the trial intervention, or if they did not receive the trial intervention they were randomised to. In seven trials these post-randomisation exclusions were balanced between treatment groups,^51^^,^^59^^,^^67^^,^^71^^,^^78^^,^^83^^,^^87^ but in two of these more than 25% of participants were excluded from the final analysis.^67^^,^^87^ In three trials more participants were excluded in the intervention group^11^^,^^56^^,^^57^ and another four trials excluded more from the control group.^12^^,^^55^^,^^60^^,^^63^ Two trials which both compared the intervention intravenous remifentanil PCA to epidural analgesia suffered more crossover from the intervention to control group rather than *vice versa*,^57^^,^^64^ meaning across these two trials 285 of 516 participants did not receive the intervention they were randomised to. Finally, one trial with four treatment arms did not include a CONSORT (Consolidated Standards of Reporting Trials) diagram, so it was unclear from which treatment groups participants were excluded.^76^

### Synthesis

#### Incidence of intrapartum fever

All included studies reported the incidence of intrapartum fever but because of heterogeneity in the trial, interventions meta-analysis of all studies was not undertaken. Instead, the trials were grouped into the following intervention types and subgroup analysis was undertaken for each: methods of reduced dose epidural, alternative methods of analgesia, prophylactic steroids, and prophylactic paracetamol. One trial with four intervention arms evaluated both an alternative method of analgesia (intravenous PCA remifentanil) and prophylactic paracetamol,^76^ so in order to avoid double counting of participants in the epidural arm, only data from the epidural alone and epidural with paracetamol arms were extracted so the results could be synthesised with a final two trials also evaluating paracetamol prophylaxis.^14^^,^^58^ Seven trials were not included in any meta-analyses because they evaluated interventions that were too clinically heterogenous to be grouped with a sufficient number of other studies.^15^^,^^51^^,^^55^^,^^59^^,^^61^^,^^65^^,^^74^ The results for primary and secondary outcomes in each individual study are shown in Supplementary file 5.

**Fig 3:**
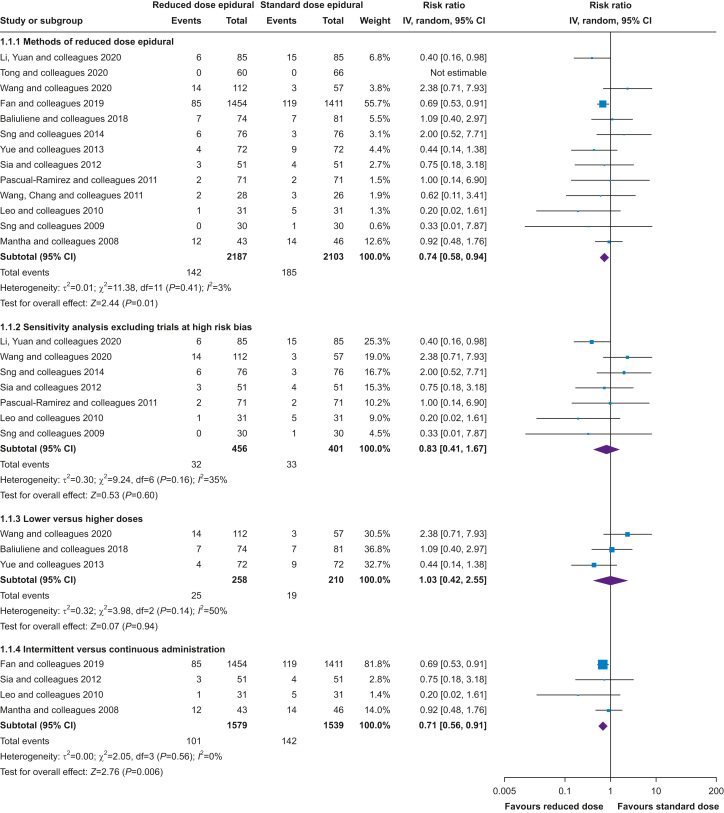
Incidence of intrapartum fever, methods of reduced dose *vs* standard dose epidural. CI, confidence interval.

Across 13 trials,^11^^,^^12^^,^^52^^,^^57^^,^^66^, ^67^, ^68^, ^69^, ^70^, ^71^, ^72^, ^73^, ^74^^,^^81^^,^^83^ consisting of a total 4290 participants, methods of reducing epidural dose reduced the incidence of intrapartum fever by 26% (RR=0.74; 95% CI, 0.58–0.94; Fig 3; analysis 1.1.1). To assess the robustness of these results, a sensitivity analysis was performed, excluding trials assessed as having a high risk of bias. Across the remaining seven studies,^66^^,^^68^^,^^72^^,^^73^^,^^81^^,^^83^ including 857 participants, methods of reducing epidural dose were not found to statistically significantly reduce the incidence of intrapartum fever as the 95% CI included a null effect (RR=0.83; 95% CI, 0.41–1.67; Fig 3; analysis 1.1.2). This sensitivity analysis removed six trials and 3515 participants^11^^,^^12^^,^^52^^,^^56^^,^^67^^,^^71^ including a large trial of 2865 participants with high risk of bias owing to differential loss to follow-up between treatment arms.^12^ Three trials with 468 participants^56^^,^^67^^,^^71^ compared different doses of local anaesthetic, and lower doses of local anaesthetic were not found to statistically significantly reduce the incidence of intrapartum fever (RR=1.03; 95% CI, 0.42–2.55; Fig 3; analysis 1.1.3), and in a further four trials, including 3118 participants,^11^^,^^12^^,^^68^^,^^73^ intermittent analgesia boluses reduced the incidence of intrapartum fever by 29% compared with continuous epidural analgesia administration (RR=0.71; 95% CI, 0.56–0.91; Fig 3; analysis 1.1.4).

**Fig 4:**
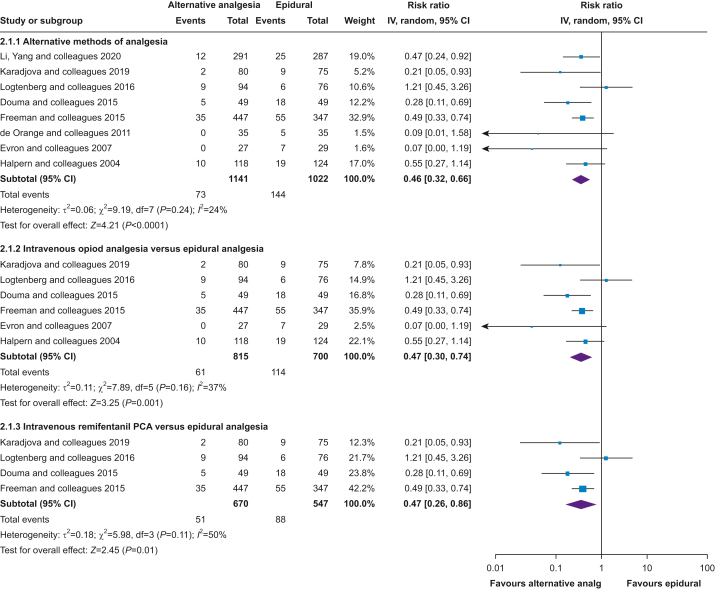
Incidence of intrapartum fever, alternative methods of analgesia. CI, confidence interval.

Eight trials,^54^^,^^57^^,^^60^^,^^64^^,^^69^^,^^78^^,^^79^^,^^83^ including 2163 participants, evaluated alternative methods of analgesia (other than epidural) and compared with epidural analgesia, alternative methods of analgesia reduced the incidence of intrapartum fever by 54% (RR=0.46; 95% CI, 0.32–0.66; Fig 4; analysis 2.1.1) and heterogeneity was not important (*I*^2^=24%). A subgroup analysis of six trials including 1515 participants^54^^,^^57^^,^^60^^,^^64^^,^^78^^,^^79^ that compared intravenous opioids with epidural analgesia suffered more heterogeneity (*I*^2^=37%) and found intravenous opioids reduced the incidence of intrapartum fever by 53% compared with epidural analgesia (RR=0.47; 95% CI, 0.30–0.74; Fig 4; analysis 2.1.2). A further subgroup analysis of the four trials and 1217 participants^54^^,^^57^^,^^60^^,^^62^ that used intravenous remifentanil PCA, found that the incidence of intrapartum fever was reduced by 53% compared with epidural analgesia (RR=0.47; 95% CI, 0.26–0.86; Fig 4; analysis 2.1.3), but the CI was wider in this analysis than that of all trials evaluating any intravenous opioid. Heterogeneity was also substantial (*I*^2^=50%).

**Fig 5:**
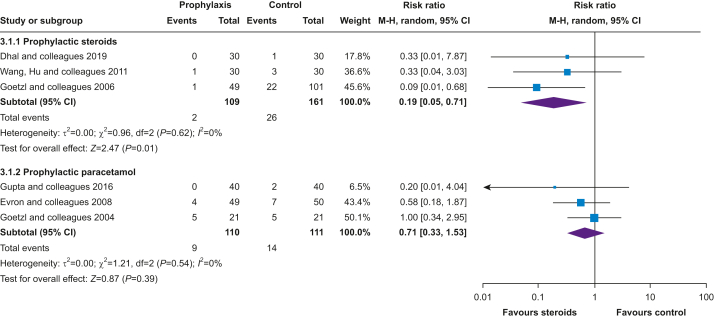
Incidence of intrapartum fever, methods of prophylaxis. CI, confidence interval.

Three trials^13^^,^^85^^,^^87^ that included a total of 270 participants found the incidence of intrapartum fever was reduced by 81% when epidural analgesia was given with prophylactic steroids, compared with if no steroids, or a placebo, were administered (RR=0.19; 95% CI, 0.05–0.71; Fig 5; analysis 3.1.1). In three trials including 221 participants,^14^^,^^58^^,^^76^ the use of prophylactic paracetamol in addition to epidural analgesia was not found to statistically significantly reduce the incidence of intrapartum fever compared with a placebo or no comparator intervention (RR=0.71; 95% CI, 0.33–1.53; Fig 5; analysis 3.1.2).

Of the remaining seven trials that were not included in any meta-analyses,^15^^,^^51^^,^^55^^,^^59^^,^^61^^,^^65^^,^^74^ only one found that the intervention under trial, in this case needle acupuncture in addition to epidural analgesia, reduced the incidence of intrapartum fever, compared with a control.^54^ Magnetic bead acupuncture,^51^ delayed administration of analgesia,^60^ the use of additional epidural opioids,^65^ prophylactic antibiotics,^15^ warming the epidural solution,^59^ or the use of a heated neck warmer^74^ were all shown to have no effect on the incidence of ERMF development.

#### Neonatal sepsis evaluation

Five studies reported the incidence of neonatal sepsis evaluation,^11^^,^^13^, ^14^, ^15^^,^^64^ only one of which reported a significant difference with high-dose steroids significantly reducing the incidence compared with a placebo (*P*=0.01).^13^ Two trials were assessed as being at low risk of bias^13^^,^^15^; Goetzl and colleagues^13^ found that prophylactic steroids statistically significantly reduced the incidence of neonatal sepsis evaluation (2/49 *vs* 18/101; *P*=0.01), compared with a placebo group, and Sharma and colleagues^15^ did not find that prophylactic antibiotics statistically significantly impacted the incidence of neonatal sepsis evaluation compared with a placebo.

#### Neonatal admission to level 2 care

Six studies reported the incidence of neonatal admission to Level 2 care,^11^^,^^13^^,^^15^^,^^60^^,^^61^^,^^74^ and a further two reported the number of neonates who were resuscitated.^54^^,^^79^ Only one trial reported a significant difference between intervention groups, with significantly more neonates of mothers who had received opioid analgesia requiring active resuscitation than those who had received epidural analgesia.^79^ Of the other trials that compared opioid with epidural analgesia that also reported data for this outcome, neither reported a significant difference.^54^^,^^60^

#### Inflammatory markers

Three different maternal inflammatory markers were reported by included studies. One reported C-reactive protein (CRP) and four reported tumour necrosis factor (TNF)-alpha, IL-6, or both. As the four studies^11^^,^^51^^,^^82^^,^^87^ evaluated three different intervention types, it is not possible to make comparisons across studies. Four studies reported a neonatal inflammatory marker, cord blood IL-6,^11–13,87^ none of which observed a statistically significant difference between intervention groups.

#### Publication bias/small study effects

The funnel plot for the incidence of intrapartum fever showed asymmetry suggesting the possibility of publication bias, although this may also reflect the clinical and statistical heterogeneity of the interventions and results (Supplementary file 6). The corresponding Egger plot (also in Supplementary file 6) should also be treated with caution as it includes subgroups of different interventions that were not formally combined in the analysis. The only subgroup with a sufficient number of trials to produce a funnel and Egger plot is methods of reduced dose epidural (Supplementary file 7), which are less suggestive of publication bias. Both funnel plots have too many mid-sized studies, and too few small studies, to justify downgrading our confidence in the findings on the use of reduced dose epidural.

### Summary of findings

The certainty of the evidence for each outcome within each comparison was variable with the majority graded as either moderate or low (Supplementary file 8, Table 1). For reduced dose epidural, alternative methods of analgesia, and prophylactic paracetamol, the certainty of evidence for incidence of intrapartum fever was graded as moderate because of the risks of bias within studies. The comparison high-dose prophylactic steroids was graded as having high certainty of evidence for incidence of intrapartum fever.

## Discussion

This systematic review evaluated methods to prevent or treat the development of ERMF. Meta-analysis suggests that reduced dose epidurals and alternative methods of analgesia may be effective at reducing the incidence of intrapartum fever. The evidence supports the effectiveness of high-dose prophylactic systemic steroids (see below), but not steroids administered epidurally, paracetamol, or antibiotics. No conclusions can be drawn regarding the impact of methods of preventing ERMF on the incidences of neonatal sepsis evaluation or admission to Level 2 care.

The secondary objectives of the review are poorly addressed. The quality of evidence for all comparisons for the incidence of neonatal sepsis evaluation and incidence of neonatal admission to Level 2 care is low. These outcomes were reported sparsely and inconsistently across comparisons, so quantitative syntheses were impossible. It is not possible to draw conclusions from the available data regarding whether preventative interventions reduce the rate of neonatal sepsis evaluation or admission to Level 2 care.

### Interpretation

The finding that reducing the dose of local anaesthetic administered by epidural analgesia may reduce the incidence of intrapartum fever is supported by the results of observational studies reporting time- and dose-dependent correlations between epidural analgesia and intrapartum fever development.^88^ Further studies have also supported the association between duration of epidural analgesia and development of intrapartum fever. Lieberman and colleagues^89^ observed that amongst women who received an epidural the incidence of fever increased from 7% for labours <6 h to 36% for labours >18 h, and Yin and Hu^90^ observed that labouring <6 h with epidural analgesia did not increase the risk of fever development, but that risk did increase significantly when epidural analgesia lasted >6 h. This evidence supports the concept that total local anaesthetic dose plays an important role in the mechanism of intrapartum fever development, as the longer an epidural is *in situ*, the higher the dose received. A higher overall dose of local anaesthetic administered could credibly cause greater release of pro-inflammatory cytokines, promoting fever. Studies specifically examining the effect of reduced local anaesthetic demonstrated a reduced incidence of fever and support this premise. Epidural analgesia of greater duration not only is associated with a *de facto* higher dose of local anaesthetic but could impair heat loss by extended sympathetic blockade, promoting the same effect, suggested elsewhere as one likely explanation for epidural hyperthermia.^18^

### Strengths and limitations

One peer reviewer expressed concern that ERMF can only occur after the administration of labour epidural analgesia. That is, it cannot occur in the absence of an epidural, and that the purpose of the article is to review ‘interventions for the prevention or treatment of epidural related maternal fever’ when ‘an epidural is already placed or is inevitable due to analgesia requirements’. On these grounds, the reviewer objected that ‘alternative methods of analgesia’ should have been included and that we should alter our eligibility criteria *post hoc*. We acknowledged these concerns, but declined to do so, citing the academic norm of maintaining prospectively agreed eligibility criteria.

The population included in this review is relatively homogenous across trials as all were either restricted to <ASA grade 3, or to healthy pregnant women, so confounding of the results owing to variations in baseline health is unlikely. The ASA classification system is used to assess an individual's preoperative anaesthesia risk and enables standardisation of health status across populations.^91^ However, pregnant women are automatically graded at least ASA 2 because of normal physiological changes and the ASA grading system is subject to significant interobserver variation in ratings, especially in obstetrics, with greatest inconsistency observed when grading healthy parturients in labour.^91^^,^^92^ This suggests that caution should be exercised in generalising the conclusions of this systematic review to maternal populations with significant co-morbidities who may have been excluded from participation in the trails under scrutiny. Also, as all trials only included participants delivering >36 weeks' gestation or at term, results of the review cannot be used to guide the management of women in premature labour. One previous review has found evidence for an association between maternal hyperthermia from any cause and neonatal morbidity, with a greater impact in pre-term infants.^18^ However, evidence to link epidural-related fever with neonatal brain injury is currently insufficient.^18^ Prematurity is an established risk factor for neonatal brain injury and although it is likely the aetiology differs to that of intrapartum fever, the risks associated with fever development may potentiate mechanisms of injury in the premature neonatal brain.^93^ Therefore, these population characteristics mean the results of our review are not generalisable to all population groups; specifically, they cannot be applied to women with pre-term labours.

The evidence in this review has several limitations, the most significant of which is the level of bias within included studies. All but seven studies were assessed as raising high or some concerns regarding risk of bias, most commonly because they failed to undertake or report intention-to-treat analyses. A large number of included studies suffered participant attrition, either through post-randomisation exclusions or deviations from intended interventions. A sensitivity analysis, excluding trials at high risk of bias, reduced the overall treatment effect of methods of reduced dose epidural, indicating that the presence of bias was likely to have influenced the results of this review. An explanation for this may be that because of inconsistent reporting across studies, results from intention-to-treat and per-protocol analyses were combined for the meta-analysis. It can be argued that strict intention-to-treat analysis is inappropriate in obstetric trials because of increased susceptibility to participant attrition and crossover between trial arms.^94^ Per-protocol or similar analysis may yield useful information in the context of an intervention which has lower analgesic effectiveness and therefore more susceptible to participant adherence and attrition from original group allocation. However, trials that deviate from an intention-to-treat can overestimate treatment effects,^95^ so it is highly likely that combining the results of intention-to-treat and per-protocol analyses has introduced bias into the quantitative synthesis.

A challenge faced by all trials involving the perinatal period is the necessary time-sensitive nature of recruitment of women who may be distressed, anxious, and in pain. Typically, women are recruited for perinatal research once they are in labour and are eligible but this has ethical implications for informed consent as women may not have enough time to understand the trial information and associated risks so may erode relationships between the caregivers and the parturient if recruitment is not handled appropriately.^96^ In regard to trials of epidural analgesia, Jackson and colleagues^97^ concluded that labouring women have an understanding of epidural risks, and this is not influenced by labour pain, anxiety, or duration of labour. However, when evaluating epidural risks the women is also anticipating a major benefit in the form of adequate pain relief which may influence the decision to provide informed consent.^96^ Two trials in this review attempted to avoid these ethical challenges of recruiting women in active labour by instead recruiting participants antenatally,^57^^,^^60^ an approach recommended by the Royal College of Obstetrics and Gynaecologists.^98^ However, this meant allocation concealment was not possible and both trials suffered participant attrition and crossover between trial arms as a consequence.

To the best of our knowledge, this is the first systematic review to have evaluated all available evidence for interventions to prevent or treat ERMF. The nature of the review question meant that the preventative and therapeutic intervention eligibility criteria was not limited to a defined set of known interventions. By consulting with an information specialist, the search strategy was updated to ensure maximum coverage of all potential interventions. This was successful as studies evaluating warming methods^59^^,^^74^ and acupuncture^51^^,^^55^ were included, two interventions that were not listed as potential preventative strategies in the review protocol. However, there are also limitations of the review process. A greater number of secondary outcomes could have been included to further evaluate the comparative effectiveness of methods of preventing or treating ERMF. Examples include maternal pain scores and satisfaction with analgesia but because of the wide range of included interventions, this was beyond the scope of the review and would have detracted focus away from the more serious neonatal consequences of ERMF.

### Recommendations for clinical practice

The evidence for alternatives to epidural analgesia reducing maternal fever has reduced relevance once a maternal decision to opt for neuraxial pain relief has been made, or the clinical circumstances recommend it. In practice, methods of reducing the overall dosage of local anaesthetic for epidural analgesia in labour whilst optimising pain control may be considered. There has been a steady progression toward low-dose epidural technique over several decades, principally driven by a desire to reduce maternal side effects such as peripheral motor blockade. The current evidence does not support the use of any additional interventions to prevent the development of ERMF. Although high-dose systemic steroids were found to be effective in reducing incidence of fever, their association with an increased incidence of neonatal bacteraemia rules them out as a safe option.^13^ Epidural analgesia remains a safe and effective method of labour analgesia, and women should continue to access this method of pain relief if they wish.

### Recommendations for further research

Further high-quality RCT evidence and mechanistic research is needed to be able to make recommendations for the appropriate management of ERMF. The current evidence base is methodologically diverse, as there is inconsistency in the assumed underlying aetiology of intrapartum fever and the type of intervention being trialled. Anti-inflammatory interventions warrant further scrutiny. The divergence in results of studies examining steroid administration suggest that higher dose steroids may offer a therapeutic intervention worth testing rigorously. However, this must be balanced against potential side effects of such doses. Studies examining the effect of paracetamol, which yielded little evidence, arguably used insufficient doses to credibly anticipate a treatment effect. Other, novel anti-inflammatory interventions are yet to be adequately examined in trials. In a retrospective study, women who received magnesium sulphate in labour, for hypertensive conditions of pregnancy, were less likely to develop fever.^99^ Parturients who developed fever were more likely to be nulliparous, have prolonged labour, receive epidural analgesia, and to require delivery by Caesarean section. Therefore this finding must be regarded in its context; an uncontrolled study, with many potential confounders. It is interesting nonetheless. Trials examining the effects of altering the temperature of anaesthetic solutions at administration are potentially promising but face many challenges to conduct in practice. Methods of reduced dose epidural, especially intermittent administration of local anaesthetic, appear to be comparatively most effective at reducing the incidence of intrapartum fever and future trials should also record neonatal outcomes including the rates of sepsis evaluation and admission to Level 2 care, to evaluate the consequences of the intervention itself, and the development of fever, on the neonate.

## Authors' contributions

Conception of review: MW

Design of the review: AC, DH, MW

Searches and screening of studies: AC

Data extraction: AC, SD, WT, HY, JW

Data analysis: AC, MB, MM

Study selection: SD

Risk of bias assessment of Chinese language studies: WT, HY, JW

Manuscript preparation: AC

Read, commented, and approved the final draft of the manuscript: DH, MM, MB, MW

## Figures and Tables

**Figure:**