# Supplementary material for: Interventions for the prevention or treatment of epidural-related maternal fever: a systematic review and meta-analysis
Source: Br J Anaesth. 2022 Aug 5;129(4):567–80. doi: 10.1016/j.bja.2022.06.022 (PMC9575042; doi:10.1016/j.bja.2022.06.022)
Supplement: Multimedia component 1 [file mmc1.docx]

All searches were carried out on 08/04/21.

No filters or limits were applied to the searches. All databases were searched from inception to search date.

MEDLINE

| Line | Search terms |
| --- | --- |
| 1 | exp fever/ or exp body temperature/ or exp inflammation/ or fever.mp or febrile.mp or heat.mp or pyrexia.mp or hyperthermia.mp or inflammation.mp or high temperature.mp |
| 2 | exp pregnancy/ or exp pregnancy complications/ or exp labor, obstetric/ or exp labor complications/ or exp delivery, obstetric/ or pregnan*.mp or intrapartum.mp or maternal.mp or maternity.mp or labour.mp or labor.mp |
| 3 | exp injections, epidural/ or exp anesthesia, epidural/ or exp analgesia, epidural/ or epidural.mp or combined spinal epidural.mp or cse.mp or neuraxial block*.mp |
| 4 | 1 and 2 and 3 |
| 5 | exp acetaminophen/ or paracetamol.mp or acetaminophen.mp or exp steroids/ or steroid*.mp or exp anti-bacterial agents/ or antibiotic*.mp or dexmedetomidine.mp or exp antipyretics/ or anti?pyretic*.mp or intervention*.mp or treatment*.mp or prevent*.mp or therap*.mp |
| 6 | epidural adj3 (intermittent or irregular or reduced dose or infrequent or alternate).mp |
| 7 | 5 or 6 |
| 8 | 4 and 7 |

EMBASE

| Line | Search terms |
| --- | --- |
| 1 | exp fever/ or exp body temperature/ or exp inflammation/ or fever.mp or febrile.mp or heat.mp or pyrexia.mp or hyperthermia.mp or inflammation.mp or high temperature.mp |
| 2 | exp pregnancy/ or exp pregnancy complication/or exp labor complication/ or exp obstetric delivery/ or pregnan*.mp or intrapartum.mp or maternal.mp or maternity.mp or labour.mp or labor.mp |
| 3 | exp epidural anesthesia/ or exp epidural analgesia/ or exp epidural drug administration/ or epidural.mp or combined spinal epidural.mp or cse.mp or neuraxial block*.mp |
| 4 | 1 and 2 and 3 |
| 5 | exp paracetamol/ or paracetamol.mp or acetaminophen.mp or exp steroid/ or steroid*.mp or exp antibiotic agent/ or antibiotic*.mp or dexmedetomidine.mp or exp antipyretic agent/ or anti?pyretic*.mp or intervention*.mp or treatment*.mp or prevent*.mp or therap*.mp |
| 6 | epidural adj3 (intermittent or irregular or reduced dose or infrequent or alternate).mp |
| 7 | 5 or 6 |
| 8 | 4 and 7 |

CINAHL

| Line | Search terms |
| --- | --- |
| 1 | MH “fever+” or MH “body temperature+” or MH “inflammation+” or fever or febrile or heat or pyrexia or hyperthermia or inflammation or high temperature |
| 2 | MH “pregnancy+” or MH “pregnancy complications+” or MH “labor complications+” or pregnan* or intrapartum or maternal or maternity or labour or labor |
| 3 | MH “injections, epidural+” or MH “analgesia, epidural” or MH “anesthesia, epidural” or MH “analgesia, obstetrical” or epidural or combined spinal epidural or cse or neuraxial block* |
| 4 | 1 and 2 and 3 |
| 5 | MH “acetaminophen” or paracetamol or acetaminophen or MH “steroids+” or steroid* or MH “antibiotics+” or MH “antibiotic prophylaxis” or antibiotic* or dexmedetomidine or anti?pyretic* or intervention* or treatment* or prevent* or therap* |
| 6 | epidural n3 (intermittent or irregular or reduced dose or infrequent or alternate) |
| 7 | 5 or 6 |
| 8 | 4 and 7 |

Web of Science

| Line | Search terms |
| --- | --- |
| 1 | TS=(fever or febrile or heat or pyrexia or hyperthermia or inflammation or high temperature) |
| 2 | TS=(pregnan* or intrapartum or maternal or maternity or labour or labor of pregnancy complication* or labor complication* or labour complication*) |
| 3 | TS=(epidural or combined spinal epidural or cse or neuraxial block* or epidural analgesia or epidural anaesthesia or epidural anesthesia) |
| 4 | 1 and 2 and 3 |
| 5 | TS=(paracetamol or acetaminophen or steroid* or antibiotic* or dexmedetomidine or anti?pyretic* or intervention* or treatment* or prevent* or therap*) |
| 6 | TS=(“epidural” near/3 intermittent or irregular or reduced dose or infrequent or alternate) |
| 7 | 5 or 6 |
| 8 | 4 and 7 |

CENTRAL

| Line | Search terms |
| --- | --- |
| 1 | exp fever/ or exp body temperature/ or exp inflammation/ or fever.mp or febrile.mp or heat.mp or pyrexia.mp or hyperthermia.mp or inflammation.mp or high temperature.mp |
| 2 | exp pregnancy/ or exp pregnancy complications/ or exp obstetric labor complications/ or exp delivery, obstetric/ or pregnan*.mp or intrapartum.mp or maternal.mp or maternity.mp or labour.mp or labor.mp |
| 3 | exp anesthesia, epidural/ or exp analgesia, epidural/ or epidural.mp or combined spinal epidural.mp or cse.mp or neuraxial block*.mp |
| 4 | 1 and 2 and 3 |
| 5 | exp acetaminophen/ or paracetamol.mp or acetaminophen.mp or exp steroids/ or steroid*.mp or exp anti-bacterial agents/ or antibiotic*.mp or dexmedetomidine.mp or exp antipyretics/ or anti?pyretic*.mp or intervention*.mp or treatment*.mp or prevent*.mp or therap*.mp |
| 6 | epidural adj3 (intermittent or irregular or reduced dose or infrequent or alternate).mp |
| 7 | 5 or 6 |
| 8 | 4 and 7 |
