# Supplementary material for: Interventions for the prevention or treatment of epidural-related maternal fever: a systematic review and meta-analysis
Source: Br J Anaesth. 2022 Aug 5;129(4):567–80. doi: 10.1016/j.bja.2022.06.022 (PMC9575042; doi:10.1016/j.bja.2022.06.022)
Supplement: Multimedia component 2 [file mmc2.docx]

Table of study characteristics

| Author, Year, Country, Multi- or Single-centre, Sample size | Population | Intervention | Comparator | Outcomes (primary, secondary) |
| --- | --- | --- | --- | --- |
| Reduced dose epidural | | | | |
| Li, Yuan et al 2020  China  Single centre  170 | Inclusion: primiparous women with singleton pregnancy, cephalic presentation, term pregnancy, require labour analgesia, ASA grade I or II, no obstetric complications, temp 36-37.5°C  Exclusion: contraindications for epidural, obstetric complication, thyroid disease, any diagnosed infection, received antibiotics with ruptured membrane over 12 hours, steroid use, received analgesia during delivery, history of mental illness | Programmed intermittent epidural analgesia at 60 minute intervals  10ml bolus of 0.075% ropivacaine + 8ml sufentanil 0.5μg/ml every 60 minutes  PCA set to 5ml bolus with lock out interval of 30 minutes | Programmed intermittent epidural analgesia at 30 minute intervals  5ml bolus of 0.075% ropivacaine + 8ml sufentanil 0.5μg/ml every 30 minutes  PCA set to 5ml bolus with lock out interval of 30 minutes | Incidence of intrapartum fever (defined >38°C), duration of each stage of labour, epidural drug usage, bleeding volume, VAS score, neonatal temperature, Apgar scores, maternal inflammatory markers |
| Tong et al  2020  China  Single centre  126 | Inclusion: full-term, singleton, cephalic position, primiparous, ASA I or II, cervical dilation of 4 cm or more  Exclusion: history of opioid usage for chronic pain, usage of opioids immediately prior to labour, contraindications for epidural analgesia | Low rate epidural: |  |  |
| Wang et al 2020  China  Single centre  180 | Inclusion: pregnant women over 18 requesting epidural during labour, cervical dilation ≤5cm, single fetus, gestational age >36 weeks, normal fetal heart rate  Exclusion: baseline numeric pain rating scale score <5, breech presentation, temp >38°C, severe preeclampsia, prenatal bleeding, ASA≥3, chronic pain, substance abuse, contraindications to epidural analgesia, allergies, BMI>40kg/m^2^, opioid use in previous 24 hours | Low dose epidural: 15ml bolus of 0.1% bupivacaine + 5 or 10μg sufentanil  Maintenance epidural 0.075% ropivacaine + 0.5μg/ml sufentanil at 8ml/hour  PCA set to 4ml bolus, lock out interval of 20 minutes | High dose epidural: 15ml bolus of 0.125% bupivacaine + 5μg sufentanil  Maintenance epidural 0.075% ropivacaine + 0.5μg/ml sufentanil at 8ml/hour  PCA set to 4ml bolus, lock out interval of 20 minutes | Analgesic onset time, mode of delivery, patient satisfaction and maternal and neonatal side effects (fever defined >38°C) |
| Fan et al 2019  China  Single centre  3000 | Inclusion: singleton, spontaneous labour, request epidural, gestation 37 to 41, nulliparous, cervical dilation 1 to 3cm  Exclusion: contraindications for epidural, baseline temp ≥37.5, allergic to opioids and/or local anaesthetics, failure to perform epidural catheterization, organic dysfunction, use of MAO inhibitors, alcohol or drug dependent, non vertex presentation, scheduled induction, multiple pregnancy, ASA 3 or higher, BM>35, high risk pregnancy | Programmed intermittent epidural analgesia at 60 minute intervals  10ml bolus of 0.08% ropivacaine + 0.4μg/ml sufentanil every 60 minutes, first bolus 75 minutes after initial loading dose  PCA set to 5ml bolus, lock out interval of 30 minutes. If pressed twice in 60 minutes an additional 5ml bolus administered. | Continuous epidural infusion  Maintenance epidural 0.08% ropivacaine + 0.4μg/ml sufentanil at 10ml/hour, initiated 15 minutes after initial loading dose  PCA set to 5ml bolus, lock out interval of 30 minutes.  If pressed twice in 60 minutes an additional 5ml bolus administered. | Incidence of intrapartum fever (defined >38°C), epidural sensory levels, VAS scores, satisfaction scores for labour epidural, epidural drug usage, number of epidural boluses, evidence of placental inflammation on histological examination, obstetric characteristics and neonate outcomes |
| Baliuliene et al 2018  Lithuania  Single centre  246 | Inclusion: healthy nulliparous women aged 18-40, singleton fetus in cephalic presentation at term who request epidural, ASA 2, cervical dilation ≤5cm and no contraindications for epidural  Exclusion: BMI >35, gestational diabetes, HTN, induced labour, PROM, fetal growth retardation, fetal abnormalities | Low dose epidural: 0.0625% bupivacaine or levobupivacaine  Maintenance epidural at 10ml/hour  PCA set to 2ml bolus, lock out interval of 15 minutes. 5ml rescue bolus administered if pain breakthrough not controlled with PCA | High dose epidural: 0.1% or 0.125% bupivacaine or levobupivacaine  Maintenance epidural at 10ml/hour  PCA set to 2ml bolus, lock out interval of 15 minutes. 5ml rescue bolus administered if pain breakthrough not controlled with PCA | Total dose of local anaesthetic used, number of episodes of breakthrough pain, satisfaction with labour analgesia, pain management efficacy, incidence of motor block, maternal or fetal side effects (fever defined ≥38°C) |
| Sng et al 2014  Singapore  Single centre  152 | Inclusion: nulliparous ASA I, ≥36 weeks gestation, requested epidural analgesia, cervical dilation ≤5cm, baseline pain score >5 on NRS, singleton fetus in vertex presentation with no pregnancy related complications  Exclusion: parenteral opioids within previous 2 hours | Computer-integrated patient controlled epidural analgesia with no basal infusion  No initial background infusion of 0.1% ropivacaine + fentanyl 0.2mcg/ml, increased to 5ml/hour if one demand PCA bolus required, 10ml/hour if two required etc. to a maximum rate 20ml/hour  PCA set to 5ml bolus, lock out interval of 10 minutes. | Computer-integrated patient controlled epidural analgesia with moderate basal infusion  Minimum background infusion of 0.1% ropivacaine + fentanyl 0.2mcg/ml at 5ml/hour, increased to 10ml/hour if one demand PCA bolus required, 15ml/hour if two required etc. to a maximum rate 20ml/hour  PCA set to 5ml bolus, lock out interval of 10 minutes | Time weighted hourly consumption of local anaesthetic, incidence of breakthrough pain, duration of labour analgesia, maternal side effects (fever defined >38.5°C) and maternal satisfaction |
| Yue et al 2013  China  Single centre  200 | Inclusion: healthy nulliparous women with a singleton cephalic presentation at term, spontaneous labour, requesting epidural analgesia  Exclusion: age <20 or >35, BMI ≥27kg/m2, baseline temperature ≥37.5°C, suspected chorioamnionitis, induced labour, history of drμg abuse, metabolic disease, pregnancy related complications, contraindications to epidural | Low dose epidural:  7ml bolus of 0.075% ropivacaine + 0.5μg/ml sufentanil every 30 minutes  No PCA  Additional 5ml bolus administered if analgesia is insufficient. | High dose epidural:  7ml bolus of 0.1% ropivacaine + 0.5μg/ml sufentanil every 30 minutes  No PCA  Additional 5ml bolus administered if analgesia is insufficient. | Incidence of intrapartum fever (defined ≥38°C) , labour events, neonate outcomes |
| Sia et al 2012  Singapore  Single centre  102 | Inclusion: ASA grade I, nulliparous parturients at term with a singleton fetus, cervical dilation <5cm, requested epidural analgesia  Exclusion: multiple pregnancy, non-cephalic presentation, obstetric complications, contraindications to neuraxial blockade, parenteral opioids in previous 2hr | Automated bolus regimen with patient controlled epidural analgesia  5ml bolus of 0.1% ropivacaine + 2μg/ml fentanyl 60 minutes after initial loading dose. Time to next bolus dependent on PCA demands.  PCA set to 5ml bolus, lock out interval of 10 minutes. Maximum dose 20ml/hour including automated boluses.  If analgesia insufficient additional 5ml bolus of 0.2% ropivacaine, and additional 50μg fentanyl if still inadequate after 10ml ropivacaine. | Continuous infusion with patient controlled epidural analgesia  Maintenance epidural 0.1% ropivacaine + 2μg/ml fentanyl at 5ml/hour  PCA set to 5ml bolus, lock out interval of 10 minutes. Maximum dose 20ml/hour including background infusion.  If analgesia insufficient additional 5ml bolus of 0.2% ropivacaine, and additional 50μg fentanyl if still inadequate after 10ml ropivacaine. | Incidence of breakthrough pain requiring additional analgesia, satisfaction with labour analgesia, obstetric and neonate outcomes, maternal side effects (fever not defined) |
| Pascual-Ramirez et al 2011  Spain  Single centre  144 | Inclusion: parturient requesting epidural analgesia, regular contractions every 2-3 mins, cervical dilation >2cm  Exclusion: fetal weight <2500g and >4500g, gestation <35 weeks, non-singleton, non-vertex presentation, ASA grade III, BMI ≥35 before pregnancy, neuromuscular disease, major complications of neuraxial procedure | Local anaesthetic epidural:  Initiated with 10ml of 0.125% or 0.25% levobupivacaine + 50μg fentanyl  Maintenance epidural 0.125% or 0.25% levobupivacaine + 0 or 2μg/ml fentanyl at 8ml/hour started immediately after initiation  No PCA  Dose dependent on anaesthetist’s judgment of the parturients clinical condition and pain | Combined spinal epidural  Initial spinal of 0.20mg morphine, 25μg fentanyl and 2.5mg hyperbaric bupivacaine.  Maintenance epidural 0.125% or 0.25% levobupivacaine + 0 or 2μg/ml fentanyl at 8ml/hour initiated at parturient request due to breakthrough pain.  No PCA  Dose dependent on anaesthetist’s judgment of the parturients clinical condition and pain | Total labour duration after analgesia initiation, obstetric outcomes and labour complications (fever not defined) |
| Wang, Chang et al 2011  China  Single centre  60 | Inclusion: healthy nulliparous women in spontaneous labour, singleton cephalic presentation at term, requesting neuraxial analgesia  Exclusion: baseline temp ≥37.5°C, metabolic disease, pregnancy complications, contraindications to neuraxial block | Delayed epidural analgesia:  Maintenance epidural 0.125% bupivacaine + 1μg/ml fentanyl at 5ml/hour only started on parturient request and with a VAS pain score >3 after spinal initiation.  PCA set to 5ml bolus, lock out interval of 15 minutes, only started on parturient request.  Additional epidural dose if analgesia insufficient after two PCA boluses. | Immediate epidural analgesia:  Maintenance epidural 0.125% bupivacaine + 1μg/ml fentanyl at 5ml/hour started immediately after spinal initiation.  PCA set to 5ml bolus, lock out interval of 15 minutes started immediately after spinal initiation.  Additional epidural dose if analgesia insufficient after two PCA boluses. | Maternal temperature change during labour (fever defined ≥38°C), obstetric and neonate outcomes |
| Leo et al 2010  Singapore  Single centre  62 | Inclusion: healthy, ASA grade I, nulliparous, >36 weeks gestation, singleton fetus in vertex presentation, cervical dilation <5cm, request epidural analgesia  Exclusion: multiple pregnancy, non cephalic presentation, obstetric complications, contraindication to neuraxial block, received parenteral opioids in previous 2hr | Automated mandatory bolus regimen:  5ml bolus 0.1% ropivacaine + 2μg/ml fentanyl every 60 minutes. If a PCA demand bolus made then the next bolus given in 30 minutes, then every 60 minutes.  PCA set to 5ml bolus, lock out interval of 10 minutes. Maximum dose 20ml/hour including automated boluses.  Additional 5ml bolus 0.2% ropivacaine if analgesia insufficient with fentanyl 50μg added if still inadequate after 10ml bolus. | Continuous background infusion:  Maintenance epidural 0.1% ropivacaine + 2μg/ml fentanyl at 5ml/hour  PCA set to 5 ml bolus, lock out interval 10 minutes. Maximum dose 20ml/hour including background infusion.  Additional 5ml bolus 0.2% ropivacaine if analgesia insufficient with fentanyl 50μg added if still inadequate after 10ml bolus. | Incidence of breakthroμgh pain requiring additonal analgesia, time-weighted local anaesthetic consumption, maternal satisfaction score, duration of effective analgesia post CSE, maternal side effects (fever not defined). |
| Sng et al 2009  Singapore  Single centre  60 | Inclusion: ASA grade I, nulliparous, term, request epidural analgesia, cervical dilation <5cm, baseline pain score >5 on VAS, singleton fetus in vertex presentation, no pregnancy related complications  Exclusion: received parenteral opioids in previous 2 hours | Computer integrated patient controlled analgesia  No initial background infusion of 0.1% ropivacaine + 2μg/ml fentanyl, increased to 5ml/hour if one demand PCA bolus made and 10ml/hour if two made etc. to a maximum of 20ml/hour. Rate reduced if no demand bolused made.  PCA set to 5ml bolus, lock out interval of 10 minutes  Additional 5ml bolus 0.2% ropivacaine if analgesia insufficient, up to an additional 20ml as required. | Patient controlled analgesia with basal infusion  Maintenance epidural 0.1% ropivacaine + 2μg/ml fentanyl at 5m/hour  PCA set to 5ml bolus, lock out interval of 10 minutes. Maximum dose 20ml/hour including background infusion.  Additional 5ml bolus 0.2% ropivacaine if analgesia insufficient, up to an additional 20ml as required. | Hourly consumption of anaesthetic solution, labour outcomes, incidence of breakthroμgh pain, maternal side effects (fever defined >38.5) |
| Mantha et al 2008  US  Single centre  92 | Inclusion: healthy nulliparous term parturients in spontaneous labour with a singleton fetus in vertex presentation  Exclusion: baseline temperature ≥37.5°C, suspected chorioamnionitis, induced labour, history of drug abuse, pregnancy related complications such as pre-eclampsia | Intermittent labour epidural analgesia initiated at parturient request.  10-15ml boluses as required of 0.125% bupivacaine + fentanyl 0.0002% or 0.1% ropivacaine + fentanyl 0.0002%  No PCA | Continuous labour epidural analgesia initiated at parturient request.  Maintenance epidural 0.125% bupivacaine + fentanyl 0.0002% or 0.1% ropivacaine + fentanyl 0.0002% at 10-15ml/hour.  No PCA | Maternal intrapartum temperature changes (fever defined >38°C), neonatal temperature and sepsis evaluation rate, maternal and cord serum cytokine changes |
| Alternative methods of analgesia | | | | |
| Li, Yang et al 2020  China  Single centre  600 | Inclusion: full term primiparous women, ASA grade I/II, age 20-35, weight 50-85kg, gestation ≥37, ability to undergo vaginal delivery  Exclusion: temperature >37°C, platelet count<90x109/L, coagulation disorder, bradycardia, puncture point infection, serious cardiopulmonary disease, liver or renal function disorder | Maintenance epidural 0.1% ropivacaine at 6ml/hour.  PCA set to 6ml bolus, lock out interval of 15 minutes. | | Intrapartum maternal fever (defined ≥38°C), VAS scores, maternal and neonatal adverse events. |
|  |  | 0.5μg/ml epidural Dexmedetomidine as part of epidural solution. | No additional intervention. |  |
| Karadjova et al 2019  North Macedonia  Single centre  155 | Inclusion: primiparous, >18 years, ASA grade I/II, singleton vertex pregnancy, gestation >36 weeks  Exclusion: complicated pregnancy, weight >120kg, contraindication for epidural, medical indication for epidural | PCA bolus doses of intravenous Remifentanil started on 0.2μg/kg, increased gradually by 0.1μg/kg up to a maximum 1μg/kg as required. Lock out interval of 2 minutes.  No background infusion. | 10ml bolus epidural 0.0625% bupivacaine + 2μg/ml fentanyl every 60 minutes.  No PCA  Additional bolus 5ml 0.1% bupivacaine if analgesia insufficient | Maternal side effects (fever defined >37°C) including oxygen saturations, neonate outcomes. |
| Logtenberg et al 2016  Netherlands  Multicentre (18 midwifery centres and 6 hospitals)  418 | Inclusion: >32 weeks gestation under the care of primary care midwives  Exclusion: younger than 18, contraindication to epidural, hypersensitivity to opioids, labour already started  Participants recruited before labour | PCA bolus doses of 30μg (solution 20μg/ml) intravenous Remifentanil, lock out intervals 3 minutes.  No background infusion.  Could increase bolus dose to 40μg if analgesia insufficient and decrease to 20μg if side effects were excessive. | Maintenance epidural 0.1% ropivacaine + sufentanil 0.5μg/ml at a variable rate defined by the anaesthetist.  No PCA  Additional bolus (dose not reported) if analgesia insufficient. | Satisfaction with pain relief, pain intensity scores, mode of delivery, maternal and neonatal outcomes (fever defined >38°C) |
| Douma et al 2015  Netherlands  Single centre  164 | Inclusion: healthy, ASA I/II, singleton pregnancy, 37-42 weeks gestation  Exclusion: BMI ≥40kg/m^2^, type 1 diabetes, preeclampsia, use of antibiotics during delivery, initial maternal oxygen saturation <98%, baseline maternal temp ≥38°C, cervical dilation >7cm, ruptured membranes >24 hr | PCA bolus dose of 40μg intravenous Remifentanil, lock out interval of 2 minutes. Maximum dose 1200μg/hour.  No background infusion.  Parturients could switch to epidural if analgesia inadequate. | Maintenance epidural 0.1% ropivacaine + 0.5μg/ml sufentanil at 10ml/hour.  No PCA  Additional 10ml bolus if analgesia insufficient. | Incidence of intrapartum fever (defined >38°C), incidence of low oxygen saturation, pain scores, maternal side effects, neonatal outcomes. |
| Freeman et al 2015  Netherlands  Multicentre (15 hospitals)  1414 | Inclusion: healthy, ASA I/II, aged over 18, scheduled to deliver vaginally after 32 weeks  Exclusion: contraindications for epidural, hypersensitivity to one of the interventions  Participants recruited before labour | PCA bolus doses of 30μg (solution 20μg/ml) intravenous Remifentanil, lock out interval 3 minutes.  No background infusion.  Could increase bolus dose to 40μg if analgesia insufficient and decrease to 20μg if side effects were excessive. | Epidural ropivacaine or bupivacaine or levobupivacaine + sufentanil or bupivacaine + fentanyl  Doses, frequency and PCA not reported. | Satisfaction with pain relief, pain intensity scores, mode of delivery, maternal and neonatal outcomes (fever defined >38°C). |
| de Orange et al 2011  Brazil  Single centre  70 | Inclusion: single fetus at term gestation, cephalic presentation, cervical dilation 3-6cm  Exclusion: fever, antibiotic use, high risk pregnancy, indication for immediate caesarean section | Non-pharmacological methods of analgesia | CSE initiated with 2.5mg 0.5% heavy bupivacaine + 5μg sufentanil when requested by the parturient.  5ml epidural bolus 0.05% bupivacaine + 0.2μg/ml sufentanil every 30 minutes, initiated after spinal analgesia. | Maternal temperature changes (fever defined ≥38°C), maternal and neonatal antibiotic use, duration of the stages of labour, need for caesarean or instrumental delivery, neonatal outcomes. |
| Evron et al 2007  Israel  Single centre  60 | Inclusion: ASA grade I/II, primiparous, spontaneous labour, singleton cephalic presentation at term  Exclusion: baseline temperature >37.4C, required caesarean delivery | PCA bolus doses of 10mg intravenous Meperidine, lock out interval of 10 minutes.  No background infusion. | Maintenance epidural 0.2% ropivacaine at 5ml/hour.  PCA set to 5ml bolus, lock out interval of 20 minutes.  Additional 10ml bolus if analgesia insufficient. | Evidence of placental infection/inflammation, maternal temperature changes (fever defined ≥38°C). |
| Halpern et al 2004  Canada  Multicentre (4 hospitals)  242 | Inclusion: nulliparous, healthy term pregnancy, gestation 37-42 weeks  Exclusion: preeclampsia, antenatal haemorrhage, BMI >35kg/m2, multiple gestation, abnormal presentation, known fetal anomalies, fetal distress | PCA bolus doses initiated with 100μg intravenous fentanyl incrementally over 1-5 minutes and then dose was adjusted to 25-50μg based on analgesia requirements and side effects. Lock out interval of 10 minutes.  No background infusion.  Offered epidural if analgesia inadequate. | PCA epidural initiated with 3 5ml boluses 0.1% bupivacaine, then additional boluses up to 25ml + 100μg fentanyl. PCA then set to 5ml bolus 0.08% bupivacaine + 1.6μg/ml fentanyl, lock out interval 10 minutes.  No background infusion.  Additional 5-10ml 0.125% bupivacaine + 50μg fentanyl if analgesia insufficient, then 5-10ml 2% lidocaine if still inadequate. | Incidence of caesarean delivery, instrumental vaginal delivery, spontaneous delivery, duration of stages of labour, maternal side effects (fever defined >38°C), neonatal outcomes. |
| Analgesia on request | | | | |
| Wassen et al 2014  Netherlands  Multicentre (2 hospitals)  493 | Inclusion: over 18 years old, pregnant with a singleton in vertex presentation, gestation >36 weeks, intention to deliver vaginally, no contraindications for epidural analgesia  Exclusion: contraindications for epidural analgesia  Some participants were recruited from 32 weeks gestation, before they went in to labour | Epidural analgesia on request (delayed initiation of analgesia)  Either intramuscular opiates or epidural depending on hospital protocol and patient preference | Routine epidural analgesia (initiated as soon as the parturient was in labour as determined by an obstetrician).  Maintenance epidural 0.125% ropivacaine or bupivacaine + 1μg/ml sufentanil at 7-10ml/hour at centre 1. At centre 2 initiated with 8ml bolus 0.180% ropivacaine + 0.5μg/ml sufentanil then PCA set to 4 ml bolus with no background infusion. | Rate of operative deliveries, maternal and labour characteristics (fever defined ≥38°C), neonatal outcomes |
| Local anaesthetic and additional opioid | | | | |
| Wang et al 2015  China  Single centre  481 | Inclusion: primiparous women with a singleton, live, term fetus, requesting epidural analgesia  Exclusion: twin gestation, multiparous, <18 or >45 years old, failed epidural or inadvertent epidural puncture, dependent or allergic to opioids, gestational diabetes and hypertension, incomplete data collection, withdrawal, violated group assignment, lost to follow up | Initial epidural bolus 10ml 0.125% ropivacaine + 0.3μg/ml sufentanil. No background infusion.  PCA set to 10-15ml bolus, lock out interval of 15 minutes. | Initial epidural bolus 10ml 0.125% ropivacaine only. No background infusion.  PCA set to 10-15ml bolus, lock out interval of 15 minutes. | Analgesic efficacy measured used the NRS scale, maternal and neonatal outcomes (fever not defined). |
| Prophylactic Steroids | | | | |
| Dhal et al 2019  India  Single centre  60 | Inclusion: ASA grade I/II, >18 years, primigravida with singleton gestation, cephalic presentation at ≥37 weeks gestation, cervical dilation ≤5cm, baseline pain score >30 on 0-100 VAS, request epidural analgesia  Exclusion: refused labour analgesia, oral or parenteral analgesics in 4hrs before neuraxial block, gestational age <37 weeks, history of obstetric complications, fetus with non-reassuring non-stress test, congenital abnormality, allergy to study drugs, diabetes, receiving steroids, history of immunosuppression, local infection, deranged clotting profile | Spinal initiated with 0.5ml 0.5% hyperbaric bupivacaine.  Maintenance epidural 0.1% levobupivacaine + 2μg/ml fentanyl at 5ml/hour.  PCA set to 5ml bolus, lock out interval of 15 minutes. | | Hourly total consumption of epidural analgesia, maternal satisfaction, pain score, maternal haemodynamics, fetal heart rate, duration of second stage of labour, mode of delivery, maternal side effects (fever not defined), neonatal outcomes |
|  |  | 8mg epidural Dexamethasone dissolved in 10ml 0.9% saline administered after epidural initiation. | 10ml 0.9% saline administered after epidural initiation. |  |
| Wang, Hu et al 2011  China  Single centre  78 | Inclusion: healthy nulliparous women in spontaneous labour, singleton in cephalic presentation, gestation >37 weeks, request epidural analgesia  Exclusion: baseline temp ≥37.5°C, metabolic disease, pregnancy related complication, contraindication to epidural | Maintenance epidural 0.125% bupivacaine + 1 μg/ml fentanyl at 5ml/hour  PCA set to 5ml bolus, lock out interval of 15 minutes.  Additional 5ml bolus if analgesia insufficient. | | Maternal temperature change (fever defined ≥38°C), obstetric and neonatal outcomes. |
|  |  | 0.2mg/ml epidural Dexamethasone combined with epidural analgesic solution | No additional intervention |  |
| Goetzl et al 2006  US  Single centre  246 | Inclusion: nulliparity, singleton gestation, gestational age ≥37 weeks, planned vaginal delivery  Exclusion: any form of diabetes mellitus, temp >99.4F on enrolment, did not elect epidural analgesia | Formulation, dose or frequency of epidural analgesia not reported. | | Rate of intrapartum fever (defined >100.4F/38°C), fetal interleukin-6 levels, neonatal bacteraemia. |
|  |  | 25mg intravenous methylprednisolone every 8 hours or 100mg intravenous methylprednisolone every 4 hours | Placebo |  |
| Prophylactic Paracetamol | | | | |
| Gupta et al 2016  India  Single centre  80 | Inclusion: primiparous ASA I/II, aged 18-35, spontaneous onset of labour at term, cervical dilation ≤5cm, single live fetus in cephalic presentation  Exclusion: participants refusal, parenteral opioids in previous 4h, systemic or local sepsis, deranged coagulation profile, multiple pregnancy, premature labour, obstetric complications, allergy to study drugs | Maintenance epidural 0.1%levobupivacaine + 2μg/ml fentanyl at 6ml/hour  PCA set to 5ml bolus, lock out interval 12 minutes | | Hourly consumption of epidural anaesthetic, pain scores, sensory and motor block, maternal haemodynamic parameters, duration of second stage of labour, mode of delivery, fetal heart rate, Apgar scores, adverse effects (fever not defined). |
|  |  | 1000mg intravenous Paracetamol in 100ml administered over 15 minutes, 30 minutes prior to epidural administration. | 100ml normal saline administered over 15 minutes, 30 minutes prior to epidural administration. |  |
| Evron et al 2008  Israel  Single centre  213 | Inclusion: healthy, singleton cephalic presentation at term, presenting in spontaneous active labour at term  Exclusion: oral temperature ≥38°C, signs of infection, ruptured membranes >24 hours, anticipated caesarean delivery | Maintenance epidural 0.2% ropivacaine at 10mg/hour.  PCA set to 10mg bolus, lock out interval 20 minutes.  Up to 4 additional 8ml bolus 0.2% ropivacaine if analgesia insufficient. | | Maternal temperature changes (fever defined ≥38°C), pain scores |
|  |  | 0.047mg/kg/min intravenous Paracetamol initiated 30 minutes after epidural. Maximum dose 2g | No additional intervention |  |
| Goetzl L, 2004  US  Single centre  42 | Inclusion: nulliparity, >37 weeks gestation, vertex presentation, able to provide informed consent, request for epidural analgesia  Exclusion: temperature >99.5F prior to epidural, pre-eclampsia, liver disease, acetaminophen allergy | Maintenance epidural 0.0625% bupivacaine + 2.5μg/cm^3^ fentanyl at 8-14cm^3^/hour.  No PCA | | Maternal temperature changes (fever defined >100.4F/38°C), obstetric and neonatal outcomes. |
|  |  | 650mg Paracetamol per rectum every 4 hours | Placebo suppository per rectum every 4 hours |  |
| Prophylactic Antibiotics | | | | |
| Sharma et al 2014  US  Single centre  400 | Inclusion: healthy nulliparous, women with singleton cephalic fetus at term, in spontaneous labour (cervical dilation 4-6cm), requesting epidural analgesia  Exclusion: not reported | Maintenance epidural 0.125% bupivacaine + 2μg/ml fentanyl at 8-10ml/hour  No PCA  2g intravenous Ampicillin every 6 hours + 1.5mg/kg intravenous Gentamicin every 8 hours if intrapartum fever develops, until afebrile for 24 hours. | | Incidence of intrapartum fever (defined ≥38°C), evidence of placental inflammation, neonatal outcomes. |
|  |  | 2g intravenous Cefoxitin before epidural initiation. Dose repeated at 6 hours post epidural if still labouring. | Intravenous saline before epidural initiation. Dose repeated at 6 hours post epidural if still labouring. |  |
| Warming methods | | | | |
| Sviggum et al 2015  US  Single centre  54 | Inclusion: healthy nulliparous women, vertex presentation fetus at term, membranes intact or ruptured <6hours  Exclusion: contraindication to epidural, allergy to local anaesthetic or opioids, increased risk of caesarean delivery, fetal abnormalities, clinical signs or symptoms of infection, baseline temp >37.6°C, cervical dilation >5cm at time of epidural request | Epidural initiated 20ml bolus 0.125% bupivacaine + 2μg/ml fentanyl over 5 minutes, then 6ml bolus every 60 minutes. No background infusion.  No PCA | | Time to achieve analgesia (VAS<3), quality of sensory blockade, body temperature (fever defined >38°C), shivering. |
|  |  | Epidural solution warmed to body temperature (37°C). | Epidural solution at room temperature (20C). |  |
| Steer 2009  UK  Single centre  70 | Inclusion: women receiving an epidural for labour analgesia, nulliparous, cervical dilation <4cm, gestation >36 weeks  Exclusion: baseline temperature >37.5°C, maternal disease including pre-eclampsia, multiple pregnancy, received steroids or NSAIDS in previous 6 hours | Epidural analgesia | | Maternal temperature rise during labour (fever defined ≥37.5°C), neonatal temperature, change in baseline FHR, Apgar scores, incidence of neonatal transfer to NICU, maternal shivering, incidence of paracetamol administration, mode of delivery, IL-6, CRP. |
|  |  | Neck collar warmed | Neck collar at room temperature |  |
| Alternative therapy | | | | |
| Wen et al  2020  China  Single centre  180 | Inclusion: age 20-40, height 150-170, weight 55-90kg, singleton of 38-42 week gestation, cephalic position, vaginal birth, ASA I, consent to study  Exclusion: histologic chorioamnionitis (HCA), premature rupture of membranes (PROM) and other pregnancy complications, upper respiratory tract or urinary tract infection in full/late term or antibiotic usage, history of sedative usage or opioid abuse, rejection of postpartum placental pathology test, prenatal fever, if analgesic used is ineffective or another analgesic used in lieu of original, emergency/prolonged labour, amniotomy (AROM), transition to C-section, usage of antibiotics during labour, if patient/family requests withdrawal from study | Epidural initiated 8ml bolus dose of ​​0.75% ropivacaine + 0.5ug/ml sufentanil then maintenance at 8ml/hour.  PCA set to 5ml bolus, lock out interval of 30 minutes. | |  |
|  |  | Auricular magnetic bead pressing. Each bead massaged for 1 minute every 20 minutes. | No additional intervention. |  |
| Xiao et al 2019  China  Single centre  127 | Inclusion: primipara, singleton fetus with cephalic presentation, gestation 37-42 weeks, age 20-35, attempting vaginal delivery, regular contractions with cervical dilation 2-3cm  Exclusion: unable to fulfil treatments, serious adverse reactions, precipitated labour, incomplete clinical data, loss to follow up | Maintenance epidural 75mg ropivacaine + 45mg sufentanil in 120ml mixed fluid at 6ml/hour.  PCA set to 8ml bolus, lock out interval of 15 minutes. | | VAS scores, obstetric complications (fever definition not reported), oxytocin use, duration of stages of labour, mode of delivery, cord blood pH, Apgar scores. |
|  |  | Electroacupuncture | No additional intervention. |  |

Abbreviations: ASA, American Society of Anesthesiology; BMI, body mass index; CSE, combined spinal epidural; FHR, fetal heart rate; NICU, neonatal intensive care unit; NRS, numerical rating scale; PCA, patient-controlled analgesia; VAS, visual analogue scale
