# Supplementary material for: Interventions for the prevention or treatment of epidural-related maternal fever: a systematic review and meta-analysis
Source: Br J Anaesth. 2022 Aug 5;129(4):567–80. doi: 10.1016/j.bja.2022.06.022 (PMC9575042; doi:10.1016/j.bja.2022.06.022)
Supplement: Multimedia component 3 [file mmc3.docx]

Table of baseline population characteristics

| Study | Age | % nulliparous | Gestation mean (SD) (weeks), except where otherwise stated | % in spontaneous labour | Baseline temperature mean (SD) (℃), expect where otherwise stated | Baseline cervical dilation mean (SD) (cm), except where otherwise stated |
| --- | --- | --- | --- | --- | --- | --- |
| Reduced dose epidural | | | | | | |
| Li, Yuan et al 2020 | 26.28 (2.31) | 100 | 39.4 (0.5) | 100 | Intervention: 36.76 (0.17)  Control: 36.75 (0.14) | NR |
| Tong et al 2020 | Intervention: 29 (5)  Control: 28 (5) | 100 | Intervention: 38.5 (0.7)  Control: 38.7 (0.7) | NR | NR | Intervention: 4.0 (0.1)  Control: 3.5 (0.3) |
| Wang et al 2020 | Intervention: 31.4 (4.3)  Control: 30.2 (3.7) | Intervention: 87  Control: 89 | Intervention: 39(36-41)  Control: 39(36-41)  Median and range | NR | NR | Intervention: 2.3 (0.7)  Control: 2.5 (0.9) |
| Fan et al 2019 | Intervention: 29 (4)  Control: 29 (4) | 100 | Intervention: 39.6 (0.9)  Control: 39.5 (0.9) | 100 | Intervention: 36.59 (0.19)  Control: 36.60 (0.21) | Intervention: 2.0 (0.8)  Control: 1.9 (0.7) |
| Baliuliene et al 2018 | Intervention: 27 (5)  Control: 27 (7)  Median and IQR | 100 | Intervention: 40 (1)  Control: 40 (1)  Median and IQR | 100 | NR | Intervention: 4 (1)  Control: 4 (0.8)  Median and IQR |
| Sng et al 2014 | Intervention: 29.5 (26-32)  Control: 30.0 (26-33)  Median and IQR | 100 | Intervention: 38 (37-39)  Control: 38 (37-39)  Median and IQR | Intervention: 39.5  Control: 48.7 | NR | Intervention: 3.0 (3.0-3.0)  Control: 3.0 (3.0-3.0)  Median and IQR |
| Yue et al 2013 | Intervention: 29.22 (2.64)  Control: 29.03 (3.03) | 100 | Intervention: 39.77 (0.97)  Control: 39.76 (0.86) | 100 | Intervention: 36.74 (0.19)  Control: 36.72 (0.15) | Intervention: 1.91 (0.39)  Control: 1.82 (0.42) |
| Sia et al 2012 | NR | 100 | NR | NR | NR | Intervention: 3.2 (0.7)  Control: 3.2 (0.9) |
| Pascual-Ramirez et al 2011 | Intervention: 31 (5)  Control: 29 (6) | Intervention: 53  Control: 53 | Intervention: 39 (2)  Control: 39 (1) | Intervention: 31  Control: 39 | NR | Intervention: 3 (2-4)  Control: 3 (2-4)  Median and IQR |
| Wang, Chang et al 2011 | Intervention: 25 (3)  Control: 25 (3) | 100 | Intervention: 39.6 (0.7)  Control: 39.1 (0.8) | 100 | Intervention: 36.9  Control: 36.8 | Intervention: 2.6 (0.2)  Control: 2.2 (0.5) |
| Leo et al 2010 | NR | 100 | NR | NR | NR | Intervention: 2.9 (0.5)  Control: 3.2 (0.69) |
| Sng et al 2009 | Intervention: 24.4 (4.4)  Control: 29.1 (4.3) | 100 | NR | NR | NR | Intervention: 3 (2-5)  Control: 3 (2-5)  Median and range |
| Mantha et al 2008 | Intervention: 26.5 (6.5)  Control: 27.0 (7.2) | 100 | Intervention: 39.4 (1.3)  Control: 39.8 (1.1) | 100 | Intervention: 36.6 (0.5)  Control: 36.7 (0.6) | Intervention: 3.5 (1.5)  Control: 3.4 (1.5) |
| Alternative methods of analgesia | | | | | | |
| Li, Yang et al 2020 | Intervention: 28.8 (2.8)  Control: 29.1 (2.9) | 100 | Intervention: 39.2 (1.1)  Control: 39.4 (1.2) | Intervention: 9.6  Control: 9.1 | Intervention: 36.6 (range 36.2-37.0)  Control: 36.6 (range 36.3-37.0) | NR |
| Karadjova et al 2019 | Intervention: 29.9 (5.2)  Control: 31.3 (3.8) | 100 | NR | NR | NR | NR |
| Logtenberg et al 2016 | Intervention: 31.7 (3.9)  Control: 31.8 (4.2) | Intervention: 68  Control: 71 | Intervention: 40.3 (1.4)  Control: 40.4 (1.6)  Median and IQR | Intervention: 81  Control: 82 | NR | Intervention: 4 (2.5)  Control: 4 (3)  Median and IQR |
| Douma et al 2015 | Intervention: 32 (4.8)  Control: 31 (5.6) | Intervention: 51  Control: 55 | Intervention: 39  Control: 40 | NR | Intervention: 37.1 (0.54)  Control: 37.2 (0.40) | Intervention: 4 (1.2)  Control: 4 (1.3) |
| Freeman et al 2015 | Intervention: 31.5 (5.1)  Control: 31.7 (4.8) | Intervention: 47  Control: 49 | Intervention: 37.8 (3.7)  Control: 37.1 (3.7)  Median and IQR | Intervention: 41  Control: 42 | NR | Intervention: 4 (2)  Control: 4 (2)  Median and IQR |
| de Orange et al 2011 | Intervention: 21.65 (18-26 range)  Control: 22.57 (18-28 range) | Intervention: 68.6  Control 68.6 | Intervention: 39.3 (0.63)  Control: 39.1 (0.63) | NR | Intervention: 36.2 (0.6)  Control: 36.2 (0.4)  Median and IQR | Intervention: 5 (1)  Control: 5 (1)  Median and IQR |
| Evron et al 2007 | Intervention: 26.4 (4.8)  Control: 25.7 (4.7) | 100 | Intervention: 39.7 (2.5)  Control: 39.6 (1.7) | 100 | NR | Intervention: 4 (1)  Control 4 (1) |
| Halpern et al 2004 | Intervention: 27 (5.8)  Control: 28 (5.4) | 100 | Intervention: 39 (1.1)  Control: 40 (0.9) | NR | NR | Intervention: 4.0 (0.9)  Control: 4.0 (0.9) |
| Analgesia on request | | | | | | |
| Wassen et al 2014 | Intervention: 30.0 (7)  Control: 30.0 (7)  Median and IQR | Intervention: 48  Control: 49 | Intervention: 40 (2)  Control: 40 (3)  Median and IQR | Intervention: 35.6  Control: 40.8 | NR | NR |
| Local anaesthetic and additional opioid | | | | | | |
| Wang et al 2015 | Intervention: 28.1 (2.7)  Control: 28.2 (2.8) | 100 | Intervention: 39.6 (0.9)  Control: 39.5 (1.1) | NR | NR | Intervention: 1.8 (0.5)  Control: 1.9 (0.5 |
| Prophylactic steroids | | | | | | |
| Dhal et al 2019 | Intervention: 24.7 (3.11)  Control: 25.9 (3.38) | 100 | NR | NR | NR | Intervention: 3.12 (0.86)  Control: 3.08 (0.77) |
| Wang, Hu et al 2011 | Intervention: 25.1 (2.7)  Control: 26.1 (3.9) | 100 | Intervention: 39.8 (0.9)  Control: 39.4 (1.0) | 100 | Intervention: 36.6  Control: 36.7 | Intervention: 2.0 (0.4)  Control: 2.0 (0.6) |
| Goetzl et al 2006 | Intervention (low dose): 21.3 (0.6)  Control: 21.9 (0.5) | 100 | Intervention (low dose): 39.3 (0.8)  Control: 39.3 (0.8) | NR | Intervention (low dose): 36.9 (0.1)  Control: 36.8 (0.1) | Intervention (low dose): 5.0 (2.0)  Control: 5.0 (2.0) |
| Prophylactic paracetamol | | | | | | |
| Gupta et al 2016 | Intervention: 26.2  Control: 25.9 | 100 | NR | 100 | NR | NR |
| Evron et al 2008 | Intervention: 27 (4)  Control: 28 (5) | Intervention: 59  Control: 56 | NR | 100 | Intervention: 36.6 (0.5)  Control: 36.6 (0.4) | Intervention: 3.0 (0.8)  Control: 2.5 (1.1) |
| Goetzl et al 2004 | Intervention: 24.6 (1.1)  Control: 21.1 (0.9) | 100 | NR | Intervention: 57  Control: 67 | Intervention: 36.8 (0.2)  Control: 36.9 (0.2) | Intervention: 4.8 (0.3)  Control: 4.5 (0.4) |
| Prophylactic antibiotics | | | | | | |
| Sharma et al 2014 | Intervention: 22 (4)  Control: 22 (4) | 100 | NR | 100 | Intervention: 36.6 (0.5)  Control: 36.7 (0.5) | Intervention: 5 (2)  Control: 4 (2)  Median and IQR |
| Warming methods | | | | | | |
| Sviggum et al 2015 | Intervention: 29.3 (4.6)  Control: 28.9 (4.4) | 100 | NR | 100 | Intervention: 36.8 (0.7)  Control: 36.7 (0.4)  Median and IQR | Intervention: 3.0 (1.0)  Control: 3.0 (1.0)  Median and IQR |
| Steer 2009 | NR | 100 | NR | NR | NR | NR |
| Alternative therapy | | | | | | |
| Wen et al 2020 | Intervention: 30.8 (5.2)  Control: 29.4 (5.2) | 100 | Intervention: 40 (39-41)  Control: 40 (39-41) | NR | Intervention: 36.9 (36.7-37.1)  Control: 36.9 (36.7-37.1)  Median and range | Intervention given at 1-2cm |
| Xiao et al 2019 | Intervention: 27.62 (2.86)  Control: 27.72 (2.60) | 100 | Intervention: 39.2 (1.1)  Control: 39.4 (1.1) | 100 | NR | NR |

Abbreviations: IQR, interquartile range; NR, not reported; SD, standard deviation
