# Supplementary material for: Interventions for the prevention or treatment of epidural-related maternal fever: a systematic review and meta-analysis
Source: Br J Anaesth. 2022 Aug 5;129(4):567–80. doi: 10.1016/j.bja.2022.06.022 (PMC9575042; doi:10.1016/j.bja.2022.06.022)
Supplement: Multimedia component 4 [file mmc4.docx]

Reasons for risk of bias judgements

| Study  Overall judgement | Reasons for judgement |
| --- | --- |
| Reduced dose epidural | |
| Li, Yuan et al 2020  Some concerns | Randomisation using random number tables  Unclear allocation process  Lacks information about deviations for interventions  No missing outcome data  ITT analysis used  Unclear whether participants, carers or outcome assessors blinded  No SAP available, unclear information about which time point was used for overall fever incidence |
| Tong et al  2020  High | Randomisation appropriate (random number tables) but allocation process unclear  Participants blinded, carers probably blinded  ITT analysis used  No missing outcome data  Outcome information only reported narratively so no information available about outcome measurement and blinding of outcome assessors  Trial protocol not located |
| Wang et al 2020  Some concerns | Randomisation and allocation process appropriate (random numbers and codes and opaque sealed envelopes, hospital pharmacy provided identical epidural solutions)  Participants, carers and outcome assessors blinded  ITT analysis used  No missing outcome data  Trial protocol (NCT03395600) did not include SAP |
| Fan et al 2019  High | Randomisation and allocation process appropriate (computer generated random numbers and sequential opaque sealed envelopes)  Participants and carers blinded  ITT analysis not used  More participants excluded post randomisation in CLEA (control) vs PIEB group  Unclear if reasons for withdrawal differed between groups  Outcome assessors blinded  Trial protocol did not include SAP |
| Baliuliene et al 2018  High | Randomisation appropriate (computer generated random numbers) but allocation process unclear  Participants, carers and outcome assessors blinded  ITT analysis not used  More participants excluded post randomisation in low dose epidural group  Trial protocol included SAP, carried out appropriately |
| Sng et al 2014  Some concerns | Randomisation and allocation process appropriate (computer generated random number tables and opaque sealed envelopes)  Participants blinded, unclear if carers and outcome assessors were: anaesthetist ‘not involved in performing block’ recorded outcome data  ITT analysis used  No missing outcome data  Trial protocol not located |
| Yue et al 2013  High | Randomisation and allocation process appropriate (computer generated random numbers and opaque sealed envelopes)  Investigators blinded, unclear whether participants and carers were  ITT analysis not used  Equal post randomisation exclusion between groups but over 25% of participants excluded  Trial protocol not located |
| Sia et al 2012  Some concerns | Randomisation and allocation process appropriate (computer generated random numbers and opaque sealed envelopes)  Participants and carers blinded  ITT analysis used  No missing outcome data  Trial protocol not located |
| Pascual-Ramirez et al 2011  Some concerns | Randomisation and allocation process appropriate (computer generated number in opaque sealed envelopes)  Some errors in reporting of baseline participants characteristics (weight in control group reported at 4.5+/-12.6kg)  Participants, carers and outcome assessors blinded  No missing outcome data  Trial protocol not located |
| Wang, Chang et al 2011  High | Randomisation and allocation process appropriate (computer generated random number codes and opaque sealed envelopes)  Participants, carers and outcome assessors unblinded  ITT analysis not used  6 participants in the delayed analgesia group delivered before they requested analgesia  Post randomisation exclusion balanced between groups  Trial protocol not located |
| Leo et al 2010  Low | Randomisation and allocation process appropriate (computer generated random number tables and sealed opaque envelopes)  Participants, carers and outcome assessors blinded  ITT analysis used  No missing outcome data  Trial protocol not located |
| Sng et al 2009  Some concerns | Randomisation and allocation process appropriate (computer generated random number tables and sealed opaque envelopes)  Participants blinded, unclear if carers and outcome assessors were  ITT analysis used  No missing outcome data  Trial protocol not located |
| Mantha et al 2008  High | Randomisation appropriate (computer programme) but allocation process unclear  Participants, carers and outcome assessors unblinded  ITT analysis not used  3 participants withdrew from intermittent analgesia (intervention) group but gave no reason, none withdrew from control group  Trial protocol not located |
| Alternative methods of analgesia | |
| Li, Yang et al 2020  Some concerns | Randomisation using random number tables  Participants and cares blinded  Unclear allocation process  ITT analysis not used  Post randomisation exclusions roughly equal between arms  22 withdrew and not included in final analysis, unclear baseline characteristics  Trial protocol did not include SAP |
| Karadjova et al 2019  Some concerns | No information on randomisation and allocation processes  Statistically significant differences in age and level of education between groups  Participants, carers and outcome assessors unblinded  ITT analysis used  No missing outcome data  Trial protocol not located |
| Logtenberg et al 2016  High | Randomisation appropriate (web based randomisation) but allocation process not concealed  Participants, carers and outcome assessors unblinded  ITT analysis not used  Switching intervention group was initially balanced but more in remifentanil group later elected epidural  More missing data in remifentanil group  Trial protocol not located |
| Douma et al 2015  High | Randomisation and allocation process appropriate (computer generated sequence and opaque sealed envelopes)  Participants, carers and outcome assessors unblinded  ITT analysis not used  Significantly more (p=0.035) more crossover from RPCA to EA compared to EA to RPCA  No missing outcome data  Trial protocol did not include SAP |
| Freeman et al 2015  High | Randomisation unclear but allocation process appropriate  Participants, carers and outcome assessors unblinded  ITT analysis not used  More individuals assigned to epidural groups elected to not receive any analgesia  More missing data from epidural group  Trial protocol did not include SAP |
| de Orange et al 2011  Some concerns | Randomisation and allocation process appropriate (computer generated random numbers and opaque sealed envelopes)  Participants, carers and outcome assessors unblinded  ITT analysis used  One participant in each group switched to the other  No missing outcome data  Trial protocol not located |
| Evron et al 2007  Some concerns | Randomisation and allocation process appropriate (computer generated codes in sequential opaque envelopes, identical syringe pumps used)  Participants blinded, unclear if carers and outcome assessors were  ITT analysis not used  Post randomisation exclusions balanced between intervention groups  Trial protocol not located |
| Halpern et al 2004  High | Randomisation and allocation process appropriate (computer generated random number system and opaque sealed envelopes)  Participants, carers and outcome assessors unblinded  ITT analysis used  More patients in opioid analgesia group crossed over to EA  No missing outcome data  Trial protocol not located |
| Analgesia on request | |
| Wassen M, 2014  Some concerns | Randomisation and allocation process appropriate (sequential opaque envelopes)  Participants, carers and outcome assessor unblinded  ITT analysis not used  No missing data  Trial protocol did not include SAP but data analysing reviewers were blind to group assignment |
| Local anaesthetic and additional opioid | |
| Wang et al 2015  High | Randomisation and allocation process appropriate (random numbers generated by online software and opaque sealed envelopes)  Participants and carers unblinded, data collectors and analysers blinded  ITT analysis not reported  32% excluded post randomisation in control group vs 24% in intervention group  Trial protocol not located |
| Prophylactic steroids | |
| Dhal et al 2019  Some concerns | Randomisation and allocation process appropriate (computer generated random numbers and opaque sealed envelopes)  Participants and carers blinded  ITT analysis used  No missing outcome data  Outcome assessors blinded  Trial protocol did not include SAP |
| Wang, Hu et al 2011  High | Randomisation and allocation process appropriate (computer generated random number codes and opaque sealed envelopes)  Participants, carers and outcome assessors blinded  ITT analysis not used  Equal post randomisation exclusion between groups but 25% of participants excluded  Trial protocol not located |
| Goetzl et al 2006  Some concerns | Randomisation and allocation process appropriate (random number generator only accessible to pharmacy staff, study medications appeared identical)  Participants, carers and outcome assessors blinded  ITT analysis used  No missing outcome data  Trial protocol not located |
| Prophylactic paracetamol | |
| Gupta et al 2016  Some concerns | Randomisation appropriate (computer generated random numbers) but allocation process unclear  Participants, carers and outcome assessors blinded  ITT analysis used  No missing outcome data  Trial protocol did not include SAP |
| Evron et al 2008  High | Randomisation and allocation process appropriate (computer generated codes in sequential opaque envelopes)  Participants, carers and outcome assessors blinded  ITT analysis not used  No CONSORT diagram and unclear which groups patients who delivered early and were therefore excluded were assigned to  Unclear what analysis was undertaken due to multiple treatment arms  Concerning lack of reporting of the results of secondary outcomes  Trial protocol not located |
| Goetzl et al 2004  Some concerns | Randomisation appropriate (computer random number generator) but allocation process unclear  Participants blinded, unclear if carers and outcome assessors were so not downgraded to low risk  ITT analysis used  No missing outcome data  Trial protocol not located |
| Prophylactic antibiotics | |
| Sharma S, 2014  Some concerns | Randomisation and allocation process appropriate (computer generated random sequence and opaque sealed envelopes), significantly more African American women in placebo group, probably due to chance  Participants blinded, unclear if carers and outcome assessors were  ITT analysis used  No missing outcome data  Trial protocol not located |
| Warming methods | |
| Sviggum H, 2015  Some concerns | Randomisation and allocation process appropriate (computer generated random numbers and opaque sealed envelopes)  Participants, carers and outcome assessors blinded  ITT analysis not used  4 participants excluded from final analysis but this was equal between groups and not likely to be due to intervention  Trial protocol not located |
| Steer 2009  High | Only a protocol registered on a trial registry was available  Randomisation and allocation process not reported in protocol  Unclear whether participants, carers and outcome assessors were blinded  ITT analysis used, but roughly 50% of participants deviated from the intervention (P Steer, personal communication, 2021 Jul 08)  No missing outcome data |
| Alternative therapy | |
| Wen et al  2020  High | Randomisation appropriate (random number tables) but allocation process unclear  Participants, carers and outcome assessors were not blinded  ITT analysis not used  Post randomisation exclusion balanced between groups  Trial protocol not located |
| Xiao et al 2018  High | Randomisation and allocation process appropriate (random number tables and sequential opaque sealed envelopes)  Participants, carers and outcome assessors unblinded  ITT analysis not used  More participants excluded post randomisation due to caesarean in control arm  6 converted to caesarean in control vs 1 in intervention  Trial protocol not located |

Abbreviations: ITT, intention-to-treat; SAP, statistical analysis plan; CONSORT, consolidated standards of reported trials; RPCA, remifentanil patient-controlled analgesia; EA, epidural analgesia
