# Supplementary material for: Interventions for the prevention or treatment of epidural-related maternal fever: a systematic review and meta-analysis
Source: Br J Anaesth. 2022 Aug 5;129(4):567–80. doi: 10.1016/j.bja.2022.06.022 (PMC9575042; doi:10.1016/j.bja.2022.06.022)
Supplement: Multimedia component 5 [file mmc5.docx]

Table of results of individual studies

| Study | Primary outcome, incidence of intrapartum fever | Secondary Outcomes | | | | | | |
| --- | --- | --- | --- | --- | --- | --- | --- | --- |
|  |  | **Incidence of neonatal admission to level 2 care** | **Incidence of neonatal sepsis evaluation** | **Neonate temperature mean (SD), or incidence of fever, as reported** | **Maternal CRP at delivery (mg/L)**  **mean (SD)** | **TNF-alpha, as reported** | **Cord blood IL-6, as reported** | **Maternal IL-6, as reported** |
| Reduced dose epidural | | | | | | | | |
| Li, Yuan et al 2020 | Intervention: 6 /85  Control: 15/85  p=0.036* | NR | NR | Intervention: 36.59 (0.34)  Control: 36.61 (0.33)  p=0.716 | Intervention: 48.42 (2.91)  Control: 53.49 (5.06)  At 2 hours post-partum | Mean (SD), ng/ml  Intervention: 2.04 (0.58)  Control: 2.30 (0.24)  At 2 hours post-partum | NR | Mean (SD), pg/ml  Intervention: 62.04 (6.49)  Control: 67.79 (6.85)  At 2 hours post-partum |
| Tong et al  2020 | Intervention: 0/60  Control: 0/66 | NR | NR | NR | NR | NR | NR | NR |
| Wang et al 2020 | Intervention: 14/112  Control: 3/57 | NR | NR | NR | NR | NR | NR | NR |
| Fan et al 2019 | Intervention: 85/1411  Control: 119/1454  p=0.007* | NR | NR | No fever (>38C) observed in either intervention group | NR | NR | Intervention: 12.4 (8.9-18.8)  Control: 10.1 (3.3-19.8)  Median and IQR  p=0.088* | NR |
| Baliuliene et al 2018 | Intervention: 7/74  Control: 7/81 | NR | NR | NR | NR | NR | NR | NR |
| Sng et al 2014 | Intervention: 6/76  Control: 3/76  p=0.494 | NR | NR | NR | NR | NR | NR | NR |
| Yue et al 2013 | Intervention: 4/72  Control: 9/72  p=0.146 | NR | NR | NR | NR | NR | NR | NR |
| Sia et al 2012 | Intervention: 3/51  Control: 4/51  p=1.0 | NR | NR | NR | NR | NR | NR | NR |
| Pascual-Ramirez et al 2011 | Intervention: 2/71  Control: 2/71  p=1.00 | NR | NR | NR | NR | NR | NR | NR |
| Wang, Chang et al 2011 | Intervention: 2/28  Control: 3/26  p=0.66 | NR | NR | NR | NR | NR | NR | NR |
| Leo et al 2010 | Intervention: 1/31  Control: 5 /31  p=1.00 | NR | NR | NR | NR | NR | NR | NR |
| Sng et al 2009 | Intervention: 0/30  Control: 1/30  p=1.000 | NR | NR | NR | NR | NR | NR | NR |
| Mantha et al 2008 | Intervention: 12/43  Control: 14/46  p=0.98 | Intervention: 2/43  Control: 0/46  p=0.45 | Intervention: 2/43  Control: 0/46  p=0.45 | Intervention: 36.8 (range 35.9-37.8)  Control: 36.7 (range 35.5-37.9) | NR | Median (IQR) pg/ml  Intervention: 1.0 (0-6.5)  Control: 1.0 (0-7.2) ^b^  At 4 hours post-partum | Intervention: 19.5 (28.0-51.2)  Control: 23.2 (12.2-40.2)^b^  Median and IQR | Median (IQR) pg/ml  Intervention: 81.1 (68.1-115.9)  Control: 111.3 (76.5-196.8) ^b^  At 4 hours post-partum |
| Alternative methods of analgesia | | | | | | | | |
| Li, Yang et al 2020 | Intervention: 12/291  Control: 25/287  p=0.024 | NR | NR | NR | NR | NR | NR | NR |
| Karadjova et al 2019 | Intervention: 2/80  Control: 9/75  p=0.028* | None resuscitated | NR | NR | NR | NR | NR | NR |
| Logtenberg et al 2016 | Intervention: 9/94  Control: 6/76  p=0.70 | NR | NR | NR | NR | NR | NR | NR |
| Douma et al 2015 | Intervention: 5/49  Control: 18/49 | NR | Intervention: 3/49  Control: 4/49 | Intervention: 2/49 ≥38C  Control: 2/49  ≥38C | NR | NR | NR | NR |
| Freeman et al 2015 | Intervention: 35/447  Control: 44/347  p<0.001*  95% CI 0.50-0.86 | Intervention: 390/687  Control: 385/671  p=0.62 | NR | NR | NR | NR | NR | NR |
| de Orange et al 2011 | Intervention: 0/35  Control: 5/35  p=0.027* | NR | NR | NR | NR | NR | NR | NR |
| Evron et al 2007 | Intervention: 0/27  Control: 7/29  p=0.02 | NR | NR | NR | NR | NR | NR | NR |
| Halpern et al 2004 | Intervention: 10/118  Control: 19/124  p=0.10 | Intervention: 61/118 active resuscitation  Control: 38/124 active resuscitation  p=0.001* | NR | Intervention: 3/118 >38C  Control: 4/124 >38C  p=0.75 | NR | NR | NR | NR |
| Analgesia on request | | | | | | | | |
| Wassen et al 2014 | Intervention: 40/255  Control: 49/233 | Intervention: 63/255  Control: 49/232 | NR | Intervention: 24/255 ≥38C  Control: 30/232 ≥38C | NR | NR | NR | NR |
| Local anaesthetic and additional opioid | | | | | | | | |
| Wang et al 2015 | Intervention:  39/182  Control: 35/164  p=0.984 | NR | NR | Intervention: 36.5 (0.2)  Control: 36.5 (0.2)  p=0.945 | NR | NR | NR | NR |
| Prophylactic steroids | | | | | | | | |
| Dhal et al 2019 | Intervention: 0/30  Control: 1/30  p=0.313 | NR | NR | NR | NR | NR | NR | NR |
| Wang, Hu et al 2011 | Intervention: 1/30  Control: 3/30  p=0.612 | NR | NR | NR | NR | Median (IQR) pg/ml  Intervention: 42.20 (79.14)  Control: 43.23 (43.93)  At delivery | Intervention: 2.37 (2.47)  Control: 4.85 (5.62)  Median and IQR  p=0.251 | Median (IQR) pg/ml  Intervention: 7.38 (12.01)  Control: 16.41 (26.13)  At delivery |
| Goetzl et al 2006 | Intervention: 1/49  Control: 22/101  p<0.001* | Intervention: 7/49  Control: 22/101 | Intervention: 2/49  Control: 18/101  p=0.01* | NR | NR | NR | Intervention (high dose): 24.0 (38.5)  Control: 32.0 (95.0)  p=0.07 (placebo vs high dose group) | NR |
| Prophylactic paracetamol | | | | | | | | |
| Gupta et al 2016 | Intervention: 0/40  Control: 2/40  p=0.15 | NR | NR | NR | NR | NR | NR | NR |
| Evron et al 2008 | Intervention: 4/49  Control: 7/50  p=0.175 | NR | NR | NR | NR | NR | NR | NR |
| Goetzl et al 2004 | Intervention: 5/21  Control: 5/21 | NR | Intervention: 7/21  Control: 4/21  p=0.48 | NR | NR | NR | NR | NR |
| Prophylactic antibiotics | | | | | | | | |
| Sharma et al 2014 | Intervention: 75/200  Control: 79/200  p=0.68 | Intervention: 0/200  Control: 1/200  p=0.32 | Intervention: 83/200  Control: 91/200  p=0.42 | Intervention: 15/200 >38C  Control: 13/300 >38C  p=0.69 | NR | NR | NR | NR |
| Warmed epidural | | | | | | | | |
| Sviggum et al 2015 | Intervention: 2/25  Control: 2/25 | NR | NR | NR | NR | NR | NR | NR |
| Warmed neck collar | | | | | | | | |
| Steer 2009 | Intervention: 5/35  Control: 4/35 | Intervention: 2/35  Control: 0/35 | NR | NR | NR | NR | NR | NR |
| Alternative therapy | | | | | | | | |
| Wen et al  2020 | Intervention: 13/81  Control: 24/79 | NR |  |  |  |  |  | Mean (SD)  pg/ml  Intervention: 30.31  Control: 19.91^c^  p<0.05*  At delivery |
| Xiao et al 2018 | Intervention: 22/60  Control: 40/60  p=0.00* | NR | NR | NR | NR | NR | NR | NR |

Abbreviations: IQR, interquartile range; ng, nanogram; NR, not reported; Pg, picogram

*Indicates statistical significance at the 5% level (p<0.05)

^a^ Reported in preliminary results in a secondary publication by Fan et al 2014 so data does not represent all participants included in the final trial

^b^ Reported graphically in a secondary publication by Mantha et al 2012

^c^ Reported graphically
