# Supplementary figures and images for: Interventions for the prevention or treatment of epidural-related maternal fever: a systematic review and meta-analysis
Source: Br J Anaesth. 2022 Aug 5;129(4):567–80. doi: 10.1016/j.bja.2022.06.022 (PMC9575042; doi:10.1016/j.bja.2022.06.022)

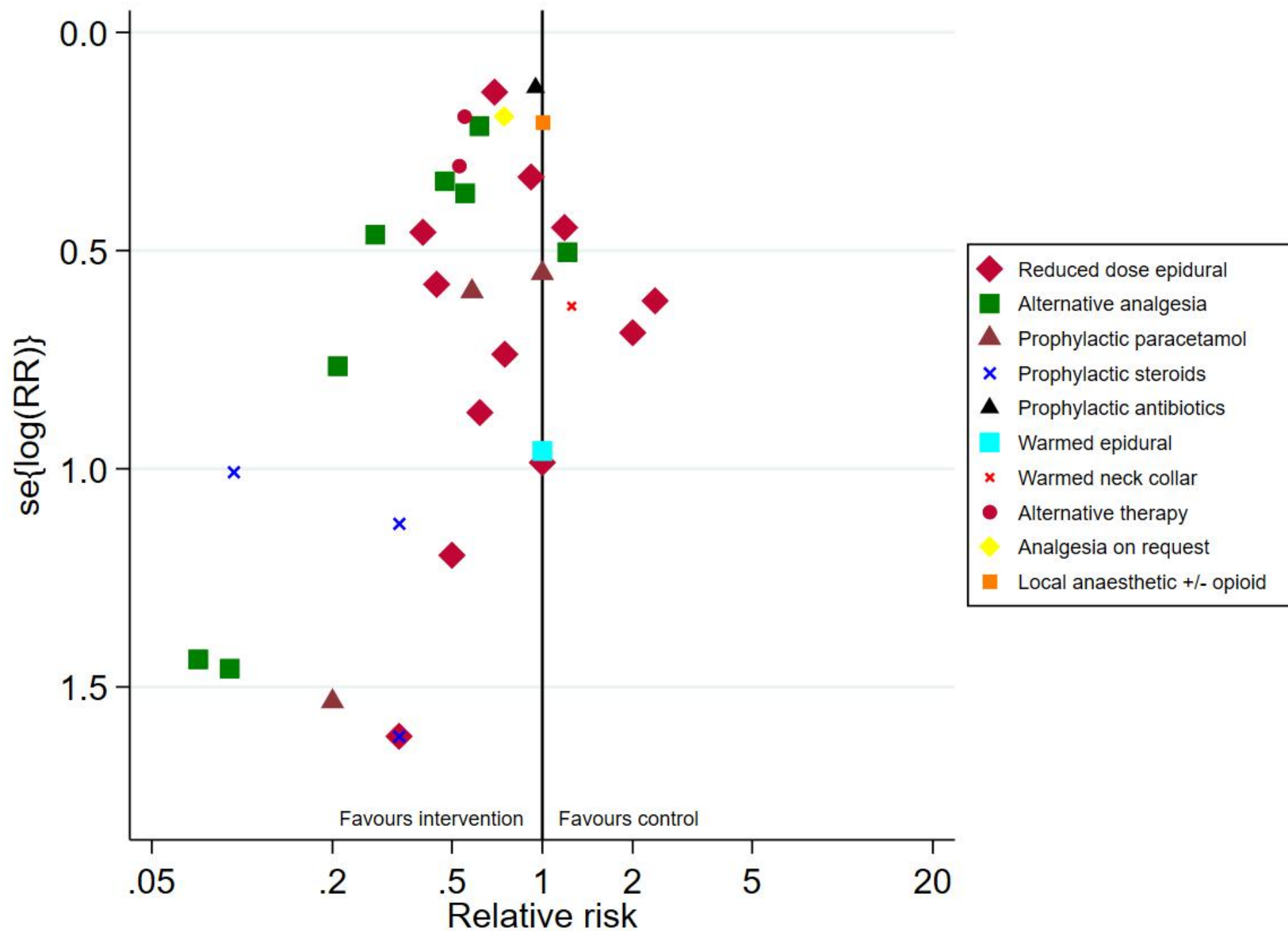

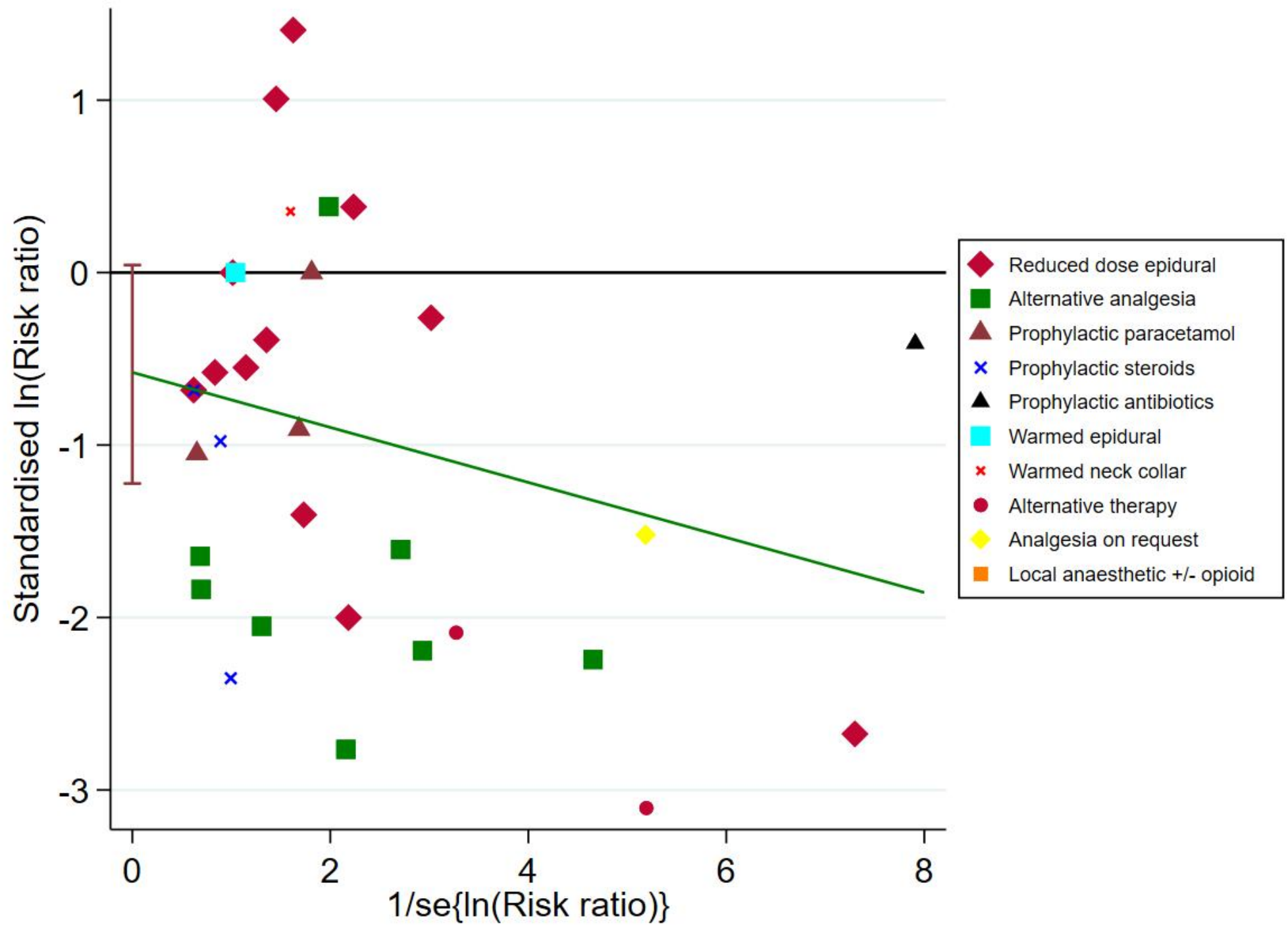

Supplement: Multimedia component 6 [file mmc6.pdf]
