# Supplementary material for: Interventions for the prevention or treatment of epidural-related maternal fever: a systematic review and meta-analysis
Source: Br J Anaesth. 2022 Aug 5;129(4):567–80. doi: 10.1016/j.bja.2022.06.022 (PMC9575042; doi:10.1016/j.bja.2022.06.022)
Supplement: Multimedia component 7 [file mmc7.docx]

Summary of evidence and GRADE tables for all comparisons

**Table I:** Summary of findings table for the comparison reduced dose epidural versus standard dose epidural to prevent ERMF

| **Outcomes** | **№ of participants  (studies)** | **Certainty of the evidence (GRADE)** | **Relative effect (95% CI)** | **Anticipated absolute effects** | |
| --- | --- | --- | --- | --- | --- |
|  |  |  |  | **Risk with standard dose epidural** | **Risk difference with reduced dose epidural** |
| Incidence of intrapartum fever | 4290 (13 RCTs) | ⨁⨁⨁◯ MODERATE ^a^ | **RR 0.74** (0.60 to 0.92) | 91 per 1,000 | **24 fewer per 1,000** (36 fewer to 7 fewer) |
| Incidence of neonatal sepsis evaluation | 89 (1 RCT) | ⨁⨁◯◯ LOW ^b,c^ | **RR 5.34** (0.26 to 108.18) | 0 per 1,000 | **0 fewer per 1,000** (0 fewer to 0 fewer) |
| Incidence of neonatal admission to level 2 care | 89 (1 RCT) | ⨁⨁◯◯ LOW ^b,c^ | **RR 5.34** (0.26 to 108.18) | 0 per 1,000 | **0 fewer per 1,000** (0 fewer to 0 fewer) |
| ***The risk in the intervention group** (and its 95% confidence interval) is based on the assumed risk in the comparison group and the **relative effect** of the intervention (and its 95% CI).  **CI:** Confidence interval; **RR:** Risk ratio | | | | | |

a. Downgraded one level due to study limitations: high risk of bias.

b. Downgraded one level due to study limitations: high risk of bias due to unclear allocation processes and lack of blinding.

c. Downgraded one level due to imprecision: low number of participants.

**Table II:** Summary of findings table for the comparison alternative methods of analgesia versus epidural to prevent ERMF

| **Outcomes** | **№ of participants  (studies)** | **Certainty of the evidence (GRADE)** | **Relative effect (95% CI)** | **Anticipated absolute effects** | |
| --- | --- | --- | --- | --- | --- |
|  |  |  |  | **Risk with epidural analgesia** | **Risk difference with alternative analgesia** |
| Incidence of intrapartum fever | 2163 (8 RCTs) | ⨁⨁⨁◯ MODERATE ^a^ | **RR 0.46** (0.32 to 0.66) | 141 per 1,000 | **76 fewer per 1,000** (96 fewer to 48 fewer) |
| Incidence of neonatal sepsis evaluation | 98 (1 RCT) | ⨁⨁◯◯ LOW ^b,c^ | **RR 0.75** (0.18 to 3.18) | 82 per 1,000 | **20 fewer per 1,000** (67 fewer to 178 more) |
| Incidence of neonatal admission to level 2 care | 1358 (1 RCT) | ⨁⨁◯◯ LOW ^b,c^ | **RR 0.99** (0.90 to 1.09) | 574 per 1,000 | **6 fewer per 1,000** (57 fewer to 52 more) |
| Incidence of neonatal resuscitation | 397 (2 RCTs) | ⨁◯◯◯ VERY LOW ^d,e,f^ | **RR 1.69** (1.23 to 2.32) | 191 per 1,000 | **132 more per 1,000** (44 more to 252 more) |
| ***The risk in the intervention group** (and its 95% confidence interval) is based on the assumed risk in the comparison group and the **relative effect** of the intervention (and its 95% CI).  **CI:** Confidence interval; **RR:** Risk ratio | | | | | |

a. Downgraded one level due to study limitations: no study assessed as being low risk of bias. Issues with lack of blinding and crossover in three trials.

b. Downgraded one level due to study limitations: high risk of bias due to lack of blinding of participants and deviations from intended intervention.

c. Downgraded one level due to imprecision: 95% CI includes null effect.

d. Downgraded one level due to study limitations: lack of blinding of participants and carers.

e. Downgraded one level due to inconsistency: one trial reported no events in either intervention arm.

f. Downgraded one level due to imprecision: low number of participants.

**Table III:** Summary of findings table for the comparison prophylactic steroids versus no steroids or placebo to prevent ERMF

| **Outcomes** | **№ of participants  (studies)** | **Certainty of the evidence (GRADE)** | **Relative effect (95% CI)** | **Anticipated absolute effects** | |
| --- | --- | --- | --- | --- | --- |
|  |  |  |  | **Risk with no steroids or placebo** | **Risk difference with prophylactic steroids** |
| Incidence of intrapartum fever | 270 (3 RCTs) | ⨁⨁⨁⨁  High | **RR 0.19** (0.05 to 0.71) | 161 per 1,000 | **131 fewer per 1,000** (153 fewer to 47 more) |
| Incidence of neonatal sepsis evaluation | 150 (1 RCT) | ⨁⨁⨁◯ MODERATE | **RR 0.23** (0.06 to 0.95) | 178 per 1,000 | **137 fewer per 1,000** (168 fewer to 9 more) |
| Incidence of neonatal admission to level 2 care | 150 (1 RCT) | ⨁⨁◯◯ LOW ^a,b^ | **RR 0.66** (0.30 to 1.43) | 218 per 1,000 | **74 fewer per 1,000** (152 fewer to 94 more) |
| ***The risk in the intervention group** (and its 95% confidence interval) is based on the assumed risk in the comparison group and the **relative effect** of the intervention (and its 95% CI).  **CI:** Confidence interval; **RR:** Risk ratio | | | | | |

a. Downgraded one level due to imprecision: 95% CI includes null effect.

b. Downgraded one level due to imprecision: low number of participants.

**Table IV:** Summary of findings table for the comparison prophylactic paracetamol versus placebo or no intervention for ERMF

| **Outcomes** | **№ of participants  (studies) Follow up** | **Certainty of the evidence (GRADE)** | **Relative effect (95% CI)** | **Anticipated absolute effects** | |
| --- | --- | --- | --- | --- | --- |
|  |  |  |  | **Risk with no paracetamol or placebo** | **Risk difference with prophylactic paracetamol** |
| Incidence of intrapartum fever | 221 (3 RCTs) | ⨁⨁◯◯ LOW ^a, b^ | **RR 0.71** (0.33 to 1.53) | 126 per 1,000 | **37 fewer per 1,000** (85 fewer to 67 more) |
| Incidence of neonatal sepsis evaluation | 42 (1 RCT) | ⨁⨁◯◯ LOW ^b, c^ | **RR 1.75** (0.60 to 5.10) | 190 per 1,000 | **143 more per 1,000** (76 fewer to 781 more) |
| ***The risk in the intervention group** (and its 95% confidence interval) is based on the assumed risk in the comparison group and the **relative effect** of the intervention (and its 95% CI).  **CI:** Confidence interval; **RR:** Risk ratio | | | | | |

a. Downgraded one level due to study limitations: high risk of bias.

b. Downgraded one level due to imprecision: 95% CI includes null effect.

c. Downgraded one level due to imprecision: low number of participants.

**Table V:** Summary of findings table for the comparison warming methods versus no warming to prevent ERMF

| **Outcomes** | **№ of participants  (studies) Follow up** | **Certainty of the evidence (GRADE)** | **Relative effect (95% CI)** | **Anticipated absolute effects** | |
| --- | --- | --- | --- | --- | --- |
|  |  |  |  | **Risk with no warming** | **Risk difference with methods of warming** |
| Incidence of intrapartum fever | 120 (2 RCTs) | ⨁⨁◯◯ LOW ^a,b^ | **RR 1.17** (0.42 to 3.27) | 100 per 1,000 | **17 more per 1,000** (58 fewer to 227 more) |
| Incidence of neonatal admission to level 2 care | 70 (1 RCT) | ⨁⨁◯◯ LOW ^a,b^ | **RR 5.00** (0.25 to 100.53) | 0 per 1,000 | **0 fewer per 1,000** (0 fewer to 0 fewer) |
| ***The risk in the intervention group** (and its 95% confidence interval) is based on the assumed risk in the comparison group and the **relative effect** of the intervention (and its 95% CI).  **CI:** Confidence interval; **RR:** Risk ratio | | | | | |

a. Downgraded one level due to study limitations: high risk of bias due to unclear randomisation and allocation and deviations from intended interventions in one trial.

b. Downgraded one level due to imprecision: 95% CI includes null effect.

**Table VI:** GRADE evidence profile for the comparison reduced dose epidural versus standard dose epidural to prevent ERMF

| **Certainty assessment** | | | | | | | **Summary of findings** | | | | |
| --- | --- | --- | --- | --- | --- | --- | --- | --- | --- | --- | --- |
| **Participants  (studies) Follow up** | **Risk of bias** | **Inconsistency** | **Indirectness** | **Imprecision** | **Publication bias** | **Overall certainty of evidence** | **Study event rates (%)** | | **Relative effect (95% CI)** | **Anticipated absolute effects** | |
|  |  |  |  |  |  |  | **With standard dose epidural** | **With reduced dose epidural** |  | **Risk with standard dose epidural** | **Risk difference with reduced dose epidural** |
| **Incidence of intrapartum fever** | | | | | | | | | | | |
| 4246 (12 RCTs) | serious ^a^ | not serious | not serious | not serious | none | ⨁⨁⨁◯ MODERATE | 188/2119 (8.9%) | 144/2127 (6.8%) | **RR 0.76** (0.61 to 0.94) | 89 per 1,000 | **21 fewer per 1,000** (from 35 fewer to 5 fewer) |
| **Incidence of neonatal sepsis evaluation** | | | | | | | | | | | |
| 89 (1 RCT) | serious ^b^ | not serious | not serious | serious ^c^ | none | ⨁⨁◯◯ LOW | 0/46 (0.0%) | 2/43 (4.7%) | **RR 5.34** (0.26 to 108.18) | 0 per 1,000 | **0 fewer per 1,000** (from 0 fewer to 0 fewer) |
| **Incidence of neonatal admission to level 2 care** | | | | | | | | | | | |
| 89 (1 RCT) | serious ^b^ | not serious | not serious | serious ^c^ | none | ⨁⨁◯◯ LOW | 0/46 (0.0%) | 2/43 (4.7%) | **RR 5.34** (0.26 to 108.18) | 0 per 1,000 | **0 fewer per 1,000** (from 0 fewer to 0 fewer) |

**CI:** Confidence interval; **RR:** Risk ratio

a. Downgraded one level due to study limitations: high risk of bias.

b. Downgraded one level due to study limitations: high risk of bias due to unclear allocation processes and lack of blinding.

c. Downgraded one level due to imprecision: low number of participants.

| **Certainty assessment** | | | | | | | **Summary of findings** | | | | |
| --- | --- | --- | --- | --- | --- | --- | --- | --- | --- | --- | --- |
| **Participants**  **(studies)**  **Follow-up** | **Risk of bias** | **Inconsistency** | **Indirectness** | **Imprecision** | **Publication bias** | **Overall certainty of evidence** | **Study event rates (%)** | | **Relative effect**  **(95% CI)** | **Anticipated absolute effects** | |
|  |  |  |  |  |  |  | **With standard dose epidural** | **With reduced dose epidural** |  | **Risk with standard dose epidural** | **Risk difference with reduced dose epidural** |
| **Incidence of intrapartum fever** | | | | | | | | | | | |
| 4290  (13 RCTs) | serious^a^ | not serious | not serious | not serious | none | ⨁⨁⨁◯  Moderate | 142/2187 (6.5%) | 185/2103 (8.8%) | RR 0.74  (0.60 to 0.92) | 91 per 1,000 | 24 fewer per 1,000  (from 36 fewer to 7 fewer) |
| **Incidence of neonatal sepsis evaluation** | | | | | | | | | | | |
| 89  (1 RCT) | serious^b^ | not serious | not serious | serious^c^ | none | ⨁⨁◯◯  Low | 0/46 (0.0%) | 2/43 (4.7%) | RR 5.34  (0.26 to 108.18) | 0 per 1,000 | 0 fewer per 1,000  (from 0 fewer to 0 fewer) |
| **Incidence of neonatal admission to level 2 care** | | | | | | | | | | | |
| 89  (1 RCT) | serious^b^ | not serious | not serious | serious^c^ | none | ⨁⨁◯◯  Low | 0/46 (0.0%) | 2/43 (4.7%) | RR 5.34  (0.26 to 108.18) | 0 per 1,000 | 0 fewer per 1,000  (from 0 fewer to 0 fewer) |

**CI:** Confidence interval; **RR:** Risk ratio

a. Downgraded one level due to study limitations: high risk of bias.

b. Downgraded one level due to study limitations: high risk of bias due to unclear allocation processes and lack of blinding.

c. Downgraded one level due to imprecision: low number of participants.

**Table VII:** GRADE evidence profile for the comparison alternative methods of analgesia versus epidural to prevent ERMF

| **Certainty assessment** | | | | | | | **Summary of findings** | | | | |
| --- | --- | --- | --- | --- | --- | --- | --- | --- | --- | --- | --- |
| **Participants  (studies) Follow up** | **Risk of bias** | **Inconsistency** | **Indirectness** | **Imprecision** | **Publication bias** | **Overall certainty of evidence** | **Study event rates (%)** | | **Relative effect (95% CI)** | **Anticipated absolute effects** | |
|  |  |  |  |  |  |  | **With epidural analgesia** | **With alternative analgesia** |  | **Risk with epidural analgesia** | **Risk difference with alternative analgesia** |
| **Incidence of intrapartum fever** | | | | | | | | | | | |
| 2163 (8 RCTs) | serious ^a^ | not serious | not serious | not serious | none | ⨁⨁⨁◯ MODERATE | 144/1022 (14.1%) | 73/1141 (6.4%) | **RR 0.46** (0.32 to 0.66) | 141 per 1,000 | **76 fewer per 1,000** (from 96 fewer to 48 fewer) |
| **Incidence of neonatal sepsis evaluation** | | | | | | | | | | | |
| 98 (1 RCT) | serious ^b^ | not serious | not serious | serious ^c^ | none | ⨁⨁◯◯ LOW | 4/49 (8.2%) | 3/49 (6.1%) | **RR 0.75** (0.18 to 3.18) | 82 per 1,000 | **20 fewer per 1,000** (from 67 fewer to 178 more) |
| **Incidence of neonatal admission to level 2 care** | | | | | | | | | | | |
| 1358 (1 RCT) | serious ^b^ | not serious | not serious | serious ^c^ | none | ⨁⨁◯◯ LOW | 385/671 (57.4%) | 390/687 (56.8%) | **RR 0.99** (0.90 to 1.09) | 574 per 1,000 | **6 fewer per 1,000** (from 57 fewer to 52 more) |
| **Incidence of neonatal resuscitation** | | | | | | | | | | | |
| 397 (2 RCTs) | serious ^d^ | serious ^e^ | not serious | serious ^f^ | none | ⨁◯◯◯ VERY LOW | 38/199 (19.1%) | 61/198 (30.8%) | **RR 1.69** (1.23 to 2.32) | 191 per 1,000 | **132 more per 1,000** (from 44 more to 252 more) |

**CI:** Confidence interval; **RR:** Risk ratio

a. Downgraded one level due to study limitations: no study assessed as being low risk of bias. Issues with lack of blinding and crossover in three trials.

b. Downgraded one level due to study limitations: high risk of bias due to lack of blinding of participants and deviations from intended intervention.

c. Downgraded one level due to imprecision: 95% CI includes null effect.

d. Downgraded one level due to study limitations: lack of blinding of participants and carers.

e. Downgraded one level due to inconsistency: one trial reported no events in either intervention arm.

f. Downgraded one level due to imprecision: low number of participants.

**Table VIII:** GRADE evidence profile for the comparison prophylactic steroids versus no steroids or placebo to prevent ERMF

| **Certainty assessment** | | | | | | | **Summary of findings** | | | | |
| --- | --- | --- | --- | --- | --- | --- | --- | --- | --- | --- | --- |
| **Participants  (studies) Follow up** | **Risk of bias** | **Inconsistency** | **Indirectness** | **Imprecision** | **Publication bias** | **Overall certainty of evidence** | **Study event rates (%)** | | **Relative effect (95% CI)** | **Anticipated absolute effects** | |
|  |  |  |  |  |  |  | **With no steroids or placebo** | **With prophylactic steroids** |  | **Risk with no steroids or placebo** | **Risk difference with prophylactic steroids** |
| **Incidence of intrapartum fever** | | | | | | | | | | | |
| 320 (3 RCTs) | not serious | not serious | not serious | serious ^a^ | none | ⨁⨁⨁◯ MODERATE | 26/161 (16.1%) | 19/159 (11.9%) | **RR 0.77** (0.45 to 1.31) | 161 per 1,000 | **37 fewer per 1,000** (from 89 fewer to 50 more) |
| **Incidence of neonatal sepsis evaluation** | | | | | | | | | | | |
| 200 (1 RCT) | not serious | not serious | not serious | very serious ^a,b^ | none | ⨁⨁◯◯ LOW | 18/101 (17.8%) | 14/99 (14.1%) | **RR 0.79** (0.42 to 1.51) | 178 per 1,000 | **37 fewer per 1,000** (from 103 fewer to 91 more) |
| **Incidence of neonatal admission to level 2 care** | | | | | | | | | | | |
| 200 (1 RCT) | not serious | not serious | not serious | very serious ^a,b^ | none | ⨁⨁◯◯ LOW | 22/101 (21.8%) | 20/99 (20.2%) | **RR 0.93** (0.54 to 1.59) | 218 per 1,000 | **15 fewer per 1,000** (from 100 fewer to 129 more) |

**CI:** Confidence interval; **RR:** Risk ratio

a. Downgraded one level due to imprecision: 95% CI includes null effect.

b. Downgraded one level due to imprecision: low number of participants.

| **Certainty assessment** | | | | | | | **Summary of findings** | | | | |
| --- | --- | --- | --- | --- | --- | --- | --- | --- | --- | --- | --- |
| **Participants**  **(studies)**  **Follow-up** | **Risk of bias** | **Inconsistency** | **Indirectness** | **Imprecision** | **Publication bias** | **Overall certainty of evidence** | **Study event rates (%)** | | **Relative effect**  **(95% CI)** | **Anticipated absolute effects** | |
|  |  |  |  |  |  |  | **With no steroids or placebo** | **With prophylactic steroids** |  | **Risk with no steroids or placebo** | **Risk difference with prophylactic steroids** |
| **Incidence of intrapartum fever** | | | | | | | | | | | |
| 270  (3 RCTs) | not serious | not serious | not serious | not serious | none | ⨁⨁⨁⨁  High | 26/161 (16.1%) | 2/109 (1.8%) | **RR 0.19**  (0.05 to 0.71) | 161 per 1,000 | **131 fewer per 1,000**  (from 153 fewer to 47 fewer) |
| **Incidence of neonatal sepsis evaluation** | | | | | | | | | | | |
| 150  (1 RCT) | not serious | not serious | not serious | serious^a^ | none | ⨁⨁⨁◯  Moderate | 18/101 (17.8%) | 2/49 (4.1%) | **RR 0.23**  (0.06 to 0.95) | 178 per 1,000 | **137 fewer per 1,000**  (from 168 fewer to 9 fewer) |
| **Incidence of neonatal admission to level 2 care** | | | | | | | | | | | |
| 150  (1 RCT) | not serious | not serious | not serious | very serious^a,b^ | none | ⨁⨁◯◯  Low | 22/101 (21.8%) | 7/49 (14.3%) | **RR 0.66**  (0.30 to 1.43) | 218 per 1,000 | **74 fewer per 1,000**  (from 152 fewer to 94 more) |

**CI:** Confidence interval; **RR:** Risk ratio

a. Downgraded one level due to imprecision: 95% CI includes null effect.

b. Downgraded one level due to imprecision: low number of participants.

**Table IX:** GRADE evidence profile for the comparison prophylactic paracetamol versus placebo or no intervention for ERMF

| **Certainty assessment** | | | | | | | **Summary of findings** | | | | |
| --- | --- | --- | --- | --- | --- | --- | --- | --- | --- | --- | --- |
| **Participants  (studies) Follow up** | **Risk of bias** | **Inconsistency** | **Indirectness** | **Imprecision** | **Publication bias** | **Overall certainty of evidence** | **Study event rates (%)** | | **Relative effect (95% CI)** | **Anticipated absolute effects** | |
|  |  |  |  |  |  |  | **With no paracetamol or placebo** | **With prophylactic paracetamol** |  | **Risk with no paracetamol or placebo** | **Risk difference with prophylactic paracetamol** |
| **Incidence of intrapartum fever** | | | | | | | | | | | |
| 221 (3 RCTs) | not serious | not serious | not serious | serious ^a^ | none | ⨁⨁⨁◯ MODERATE | 14/111 (12.6%) | 9/110 (8.2%) | **RR 0.71** (0.33 to 1.53) | 126 per 1,000 | **37 fewer per 1,000** (from 85 fewer to 67 more) |
| **Incidence of neonatal sepsis evaluation** | | | | | | | | | | | |
| 42 (1 RCT) | not serious | not serious | not serious | very serious ^a,b^ | none | ⨁⨁◯◯ LOW | 4/21 (19.0%) | 7/21 (33.3%) | **RR 1.75** (0.60 to 5.10) | 190 per 1,000 | **143 more per 1,000** (from 76 fewer to 781 more) |

**CI:** Confidence interval; **RR:** Risk ratio

a. Downgraded one level due to imprecision: 95% CI includes null effect.

b. Downgraded one level due to imprecision: low number of participants.

| **Certainty assessment** | | | | | | | **Summary of findings** | | | | |
| --- | --- | --- | --- | --- | --- | --- | --- | --- | --- | --- | --- |
| **Participants**  **(studies)**  **Follow-up** | **Risk of bias** | **Inconsistency** | **Indirectness** | **Imprecision** | **Publication bias** | **Overall certainty of evidence** | **Study event rates (%)** | | **Relative effect**  **(95% CI)** | **Anticipated absolute effects** | |
|  |  |  |  |  |  |  | **With no paracetamol or placebo** | **With prophylactic paracetamol** |  | **Risk with no paracetamol or placebo** | **Risk difference with prophylactic paracetamol** |
| **Incidence of intrapartum fever** | | | | | | | | | | | |
| 221  (3 RCTs) | serious^a^ | not serious | not serious | serious^b^ | none | ⨁⨁◯◯  Low | 14/111 (12.6%) | 9/110 (8.2%) | **RR 0.71**  (0.33 to 1.53) | 126 per 1,000 | **37 fewer per 1,000**  (from 85 fewer to 67 more) |
| **Incidence of neonatal sepsis evaluation** | | | | | | | | | | | |
| 42  (1 RCT) | not serious | not serious | not serious | very serious^b,c^ | none | ⨁⨁◯◯  Low | 4/21 (19.0%) | 7/21 (33.3%) | **RR 1.75**  (0.60 to 5.10) | 190 per 1,000 | **143 more per 1,000**  (from 76 fewer to 781 more) |

**CI:** confidence interval; **RR:** risk ratio

a. Downgraded one level due to study limitations: high risk of bias.

b. Downgraded one level due to imprecision: 95% CI includes null effect.

c. Downgraded one level due to imprecision: low number of participants.

**Table X:** GRADE evidence profile for the comparison warming methods versus no warming to prevent ERMF

| **Certainty assessment** | | | | | | | **Summary of findings** | | | | |
| --- | --- | --- | --- | --- | --- | --- | --- | --- | --- | --- | --- |
| **Participants  (studies) Follow up** | **Risk of bias** | **Inconsistency** | **Indirectness** | **Imprecision** | **Publication bias** | **Overall certainty of evidence** | **Study event rates (%)** | | **Relative effect (95% CI)** | **Anticipated absolute effects** | |
|  |  |  |  |  |  |  | **With no warming** | **With methods of warming** |  | **Risk with no warming** | **Risk difference with methods of warming** |
| **Incidence of intrapartum fever** | | | | | | | | | | | |
| 120 (2 RCTs) | serious ^a^ | not serious | not serious | serious ^b^ | none | ⨁⨁◯◯ LOW | 6/60 (10.0%) | 7/60 (11.7%) | **RR 1.17** (0.42 to 3.27) | 100 per 1,000 | **17 more per 1,000** (from 58 fewer to 227 more) |
| **Incidence of neonatal admission to level 2 care** | | | | | | | | | | | |
| 70 (1 RCT) | serious ^a^ | not serious | not serious | serious ^b^ | none | ⨁⨁◯◯ LOW | 0/35 (0.0%) | 2/35 (5.7%) | **RR 5.00** (0.25 to 100.53) | 0 per 1,000 | **0 fewer per 1,000** (from 0 fewer to 0 fewer) |

**CI:** Confidence interval; **RR:** Risk ratio

a. Downgraded one level due to study limitations: high risk of bias due to unclear randomisation and allocation and deviations from intended interventions in one trial.

b. Downgraded one level due to imprecision: 95% CI includes null effect.
